# Cancer cell lipid class homeostasis is altered under nutrient-deprivation but stable under hypoxia

**DOI:** 10.1186/s12885-019-5733-y

**Published:** 2019-05-28

**Authors:** Jan Lisec, Carsten Jaeger, Rida Rashid, Rimsha Munir, Nousheen Zaidi

**Affiliations:** 1 Bundesanstalt für Materialforschung und -prüfung (BAM), Department of Analytical Chemistry, Richard-Willstätter-Straße 11, 12489 Berlin, Germany; 2 Charité - Universitätsmedizin Berlin, Molekulares Krebsforschungszentrum (MKFZ), Augustenburger Platz 1, 13353 Berlin, Germany; 3 Berlin Institute of Health (BIH), Anna-Louisa-Karsch-Straße 2, 10178 Berlin, Germany; 4 Cancer Biology Lab, MMG, University of the Punjab, Lahore, Pakistan

**Keywords:** Tumor metabolism, Fatty acid metabolism, Lipidomic profile, Metabolic stress

## Abstract

**Background:**

Cancer cells modify the balance between fatty acid (FA) synthesis and uptake under metabolic stress, induced by oxygen/nutrient deprivation. These modifications were shown to alter the levels of individual triglyceride (TG) or phospholipid sub-species. To attain a holistic overview of the lipidomic profiles of cancer cells under stress we performed a broad lipidomic assay, comprising 244 lipids from six major classes. This assay allowed us to perform robust analyses and assess the changes in averages of broader lipid-classes, stratified on the basis of saturation index of their fatty-acyl side chains.

**Methods:**

Global lipidomic profiling using Liquid Chromatography-Mass Spectrometry was performed to assess lipidomic profiles of biologically diverse cancer cell lines cultivated under metabolically stressed conditions.

**Results:**

Neutral lipid compositions were markedly modified under serum-deprived conditions and, strikingly, the cellular level of triglyceride subspecies decreased with increasing number of double bonds in their fatty acyl chains. In contrast and unexpectedly, no robust changes were observed in lipidomic profiles of hypoxic (2% O_2_) cancer cells despite concurrent changes in proliferation rates and metabolic gene expression.

**Conclusions:**

Serum-deprivation significantly affects lipidomic profiles of cancer cells. Although, the levels of individual lipid moieties alter under hypoxia (2% O_2_), the robust averages of broader lipid classes remain unchanged.

**Electronic supplementary material:**

The online version of this article (10.1186/s12885-019-5733-y) contains supplementary material, which is available to authorized users.

## Background

Lipid metabolism has emerged as an important aspect of cancer cell metabolism and is widely shown to be associated with various malignant processes [1–3]. Cancer cells require a constant supply of lipids for membrane biogenesis [4] and protein modifications [5]. In addition to that, lipids are also involved in energy supply via β oxidation of fatty acids and for the biosynthesis of various protumorigenic lipid-signaling molecules [6]. Several studies have shown that, in order to cope with these increased demands, cancer cells activate de novo lipid synthesis pathways [4, 6–9]. Fatty acid synthase (FASN) –a key-regulator of de novo fatty acid (FA) synthesis– has been extensively shown to fuel cancer cell proliferation and malignant progression [3]. Expression of 3-hydroxy-3-methylglutaryl-CoA reductase (HMGCR) –the rate-controlling enzyme of the mevalonate pathway– is also up-regulated in cancers [10]. Importantly, inhibition of FA synthesis or cholesterol synthesis pathways results in growth-arrest of lipogenic tumor cells rendering these pathways interesting targets for antineoplastic therapy [4, 11–17]. Although endogenous FA synthesis has historically been considered the principal source of fatty acids (FAs) in cancer cells, lipolytic phenotypes are also widely recognized (reviewed in) [6]. For example, it has been reported that in addition to the markers of de novo synthesis (FASN) different cancer cells also express markers of lipolysis (lipoprotein lipase, LPL) and exogenous FA uptake (CD36) [9].

Cancer cells are often exposed to a metabolically challenging environment, with scarce availability of oxygen and nutrients. This metabolic stress leads to changes in the balance between the endogenous synthesis, and the exogenous uptake of fatty acids [3, 18, 19]. These alterations in FA acquisition mode may significantly affect the lipidomic profiles of cancer cells. Mammalian cells have a limited ability to synthesize polyunsaturated fatty acids de novo, as they lack the Δ12 desaturase [20]. Therefore, enhanced de novo FA synthesis enriches the cancer cell membranes with saturated and/or mono-unsaturated fatty acids [21].

Although several groups have studied regulation of FA metabolism under metabolic stress, only a few have investigated their lipidomic profiles [22–25]. Moreover, most studies on the impact of metabolic stress on lipidomic profiles of cancer cells are limited to specific lipid classes. For instance, it was shown that under hypoxia breast cancer cells display modified phospholipid profiles mainly characterized by the presence of shorter and more saturated acyl chains while other lipid classes were not considered [26]. Another study reported that under hypoxic conditions cellular levels of triglycerides with three double bonds were significantly decreased in MCF7 breast cancer cells [19], but significantly increased in U87 glioblastoma cells, but data on membrane lipids were not collected. A recent work by *Ackerman* et al [27] studied the impact of serum/oxygen deprivation on various lipid classes in renal cancer cells. They reported that serum-deprivation with/without hypoxia affects triglyceride composition in these cells with significant decrease in the abundance of unsaturated triglycerides and a shift toward triglyceride saturation.

Herein, to study the complex interplay between metabolic stress and lipid metabolism in cancer cells, we selected a biologically diverse panel of cancer cell lines –three leukemia cell lines, two colon cancer cell lines and one lung cancer cell line. We were mainly interested in studying the impact of physiologically relevant metabolic stress on lipidomic profiles of cancer cells. To achieve that cancer cells were cultivated under nutrient-deprivation and/or hypoxia [28, 29]. In order to gain more systematic insight on the effects of metabolic stress on lipidomic profiles we performed a broad lipidomics assay comprising 244 lipids from six major classes. To this end we identified multiple changes in lipidomic profiles of cancer cells cultivated under low-serum or lipid-deficient conditions. Interestingly, no robust changes were observed in lipidomic profiles of hypoxic cancer cells indicating that the cells maintain lipid class homeostasis.

## Methods

### Cell culture and treatments

The SW480, SW620, A549, KG1, KCL22 and KU812 cell lines were purchased from American Type Culture Collection and were maintained in DMEM (Gibco, 31,966–021) or RPMI 1640 medium (Gibco, 61,870–010) media supplemented with 10% fetal bovine serum (FBS) (Sigma, F75240) and penicillin-streptomycin solution (Corning, 30–002-CI). Cell cultures were maintained in the atmosphere of 5% CO_2_ and 37 °C. For all experiments cells were initially seeded and cultivated in normal media for 24 h. Then to induce metabolic stress media and/or growth conditions were respectively changed and cells were cultivated for additional 48 h under either one of the following condition: lipoprotein deficient medium (LPDS serum), low-serum (LS) medium (2% serum), hypoxia (2% O2), or hypoxia in combination with LS medium. For lipoprotein deficient conditions the media were supplemented with lipoprotein deficient serum (LPDS) that was purchased from Merck (LP4) and used according to manufacturer’s guidelines. For determining the cells number cells were stained with trypan blue and counted using Countess® automated cell counter (Invitrogen). Cell lines were commercially authenticated (Eurofins, Germany) and mycoplasma tested prior to submission of this manuscript.

### Quantitative RT-PCR

For quantitative RT-PCR, total RNA was extracted from cell pellets using Quick-RNA™ MiniPrep Plus (Zymo Research). All RNA samples were reverse-transcribed into cDNA using SuperScript™ III Reverse Transcriptase (Thermo Scientific, 18,080,093) and Oligo(dT)18 Primers (Thermo Scientific, SO131). Quantitative PCR was performed using a TaqMan™ Gene Expression Master Mix (4,369,016, Applied Biosystems) *via*StepOne Real-Time PCR Systems (Applied Biosystems). The TaqMan Gene Expression assays used were Hs01005622_m1 (fatty acid synthase, FASN), Hs00168352_m1 (3-hydroxy-3-methylglutaryl-CoA reductase, HMGCR), Hs00996004_m1 (monoglyceride lipase, MGLL), Hs00173425_m1 (lipoprotein lipase, LPL) and Hs00354519_m1 (CD36). The expression of each gene was normalized to the expression of GADPH (Hs02786624_g1).

### Lipid extractions

First, the cell pellets were washed with 0.5 mL 0.9% NaCl. For extraction of lipids the pellets were resuspended in 1 ml ice-cold MMC (1:1:1 v/v/v methanol/MTBE/chloroform). Samples were incubated on an ultrasonic bath for 2 min. Phase separation was induced by adding 300 μL MS-grade water. After 10 min incubation, the samples were centrifuged for 10 min at 1000 rpm and the upper (organic) phase was collected. Then 200 μL of collected organic phase were dried in a vacuum rotator and stored at − 20 °C until analysis.

### Determination of Total triglyceride and cholesterol Ester contents

Total triglyceride content in the lipid extracts was spectrophotometrically determined using commercially available kit (*Analyticon Biotechnologies AG, Catalogue # 5052)* against a calibration-curve generated using known concentrations of triglyceride standard (*SUPELCO, 17811-1AMP).* Cholesteryl esters were quantified using *Cholesterol/ Cholesteryl Ester Quantitation Kit* (*Abcam, ab65359*) was used according to manufacturer’s guidelines.

### Lipidomic profiling and data processing

Dried sample extracts were reconstituted in 100 μL 2:1:1 v/v/v isopropanol/acetonitrile/water. 5 μL aliquots were injected into an ACQUITY I-class ultra-performance liquid chromatography (UPLC) system (Waters, Germany) coupled to an Impact II high-resolution quadrupole time-of-flight mass spectrometer (BrukerDaltonik GmbH, Germany). Chromatographic separation was achieved by gradient elution (%A: 0 min, 60; 1.2 min, 57; 1.26 min, 50; 7.2 min, 46; 7.26 min, 30; 10.8 min, 0; 12.96 min, 0; 13.02 min, 60; 14.4 min, 60) using a buffered solvent system (A: 60:40 v/v acetonitrile/water, B: 90:10 v/v isopropanol:water, both with 10 mM ammonium formate and 0.1% formic acid) and a 2.1 mm × 75 mm × 1.7 μm CSH-C18 column (Waters, Germany) equipped with an 0.2 μm pre-column in-line filter. The flow rate was 0.5 mL min^− 1^ and column temperature was 55 °C. Electrospray ionization (ESI) conditions were as follows: polarity (+), capillary voltage, 4500 V, end plate offset, 500 V, nebulizer pressure, 2.5 bar, dry gas (N2) flow, 8 L/min. Ion transfer parameters were set to: Funnel 1 RF, 200 Vpp, Funnel 2 RF, 200 Vpp, Hexapole RF, 50 Vpp, Quadrupole Ion Energy, 5 eV, Low Mass, 100 m/z, Collision Energy, 8.0 eV, Pre Pulse Storage, 6.0 μs, Stepping Mode, Basic, Collision RF, 500–1000 Vpp, Transfer Time, 60–100 μs, Timing, 50/50, Collision Energy, 100–250%. Alternating MS and MS/MS scans were acquired using a Sequential Windowed Acquisition of All Theoretical Fragment Ion Mass Spectra (SWATH) scheme (m/z 350–975, width 25 Da). For internal calibration, Na formate clusters were spiked into the LC effluent at the end of each run.

After data acquisition, files were converted to Analysis Base File (ABF) format using a publicly available converter (Reifycs, Japan) and imported into MS-DIAL (Tsugawa et al. 2015). MS-DIAL parameter settings were as follows: Soft Ionization, Data independent MS/MS, Centroid data, Positive ion mode, Lipidomics. Detailed analysis settings were left at default, except for Retention time end (10 min), Alignment Retention time tolerance (0.2 min), Identification Retention time tolerance (3 min) and Identification score cut off (60%). Identified peaks were exported to a text file and subjected to statistical analysis.

Saturation indices were calculated as a ratio of total saturated fatty acids to total unsaturated fatty acids [27].The total level of saturated fatty acids in individual lipid class was calculated by summing up the intensities of each saturated fatty acid containing lipid multiplied by the number of saturated fatty acids present in that particular lipid moiety. The total level of un-saturated fatty acids in individual lipid class was calculated by summing up the intensities of each un-saturated fatty acid containing lipid multiplied by the number of un-saturated fatty acids present in that particular lipid moiety.

### Statistical analysis

The differences between groups were analyzed by ANOVA or t-test (paired or unpaired), where applicable. Statistical analyses and graphical representations for lipidomic data and quantitative RT-PCR data were performed using the R software environment 3.4.2 (http://cran.r-project.org/) or MetaboAnalyst 3.5 (http://www.metaboanalyst.ca/faces/home.xhtml). *P*-values < 0.05 were considered statistically significant and indicated when different.

## Results

### Comparison of baseline lipidomic profiles of cancer cell lines

**Fig. 1:**
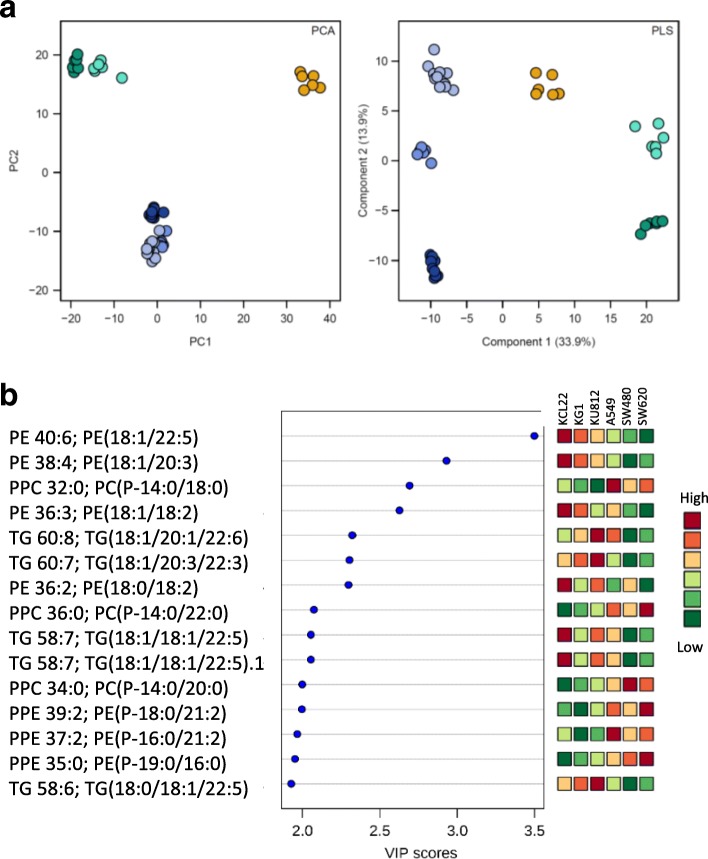
Baseline lipidomic profiles in selected cancer cell lines. **a** *(Left- panel)* Principal component analysis (PCA) of lipidomic profiles KCL22 (Leukemia), KG1 (Leukemia), KU812 (Leukemia), SW480 (Colon cancer), SW620 (Colon cancer) and A549 (Lung Cancer) cell lines at baseline level. Percentage of the variance captured by each principal component (PC) is given close to each respective axis. *(Right-panel)* PLS-DA model analysis of 244 common lipid molecules to differentiate six different cell lines (i.e. KG1, KCL22, KU812, SW480, SW620 and A549) (**b**) Potential discriminatory lipid molecules identified through VIP scores (VIP values of > 2.0) derived from PLS-DA modeling of complete data matrix. Resulting VIP scores for top 15 lipid molecules are shown in increasing order of VIP score values to highlight their discriminatory potential

First, we compared the baseline lipidomic profiles of the selected cell lines. Global lipidomic profiling using Liquid Chromatography-Mass Spectrometry (LC-MS) followed by automatic annotation using MS-Dial (v.2.72 [30]) allowed to detect 244 lipid compounds each present in at least 90% of all samples (Table 1). Majority of the detected lipid molecules were either phospholipids (*n* = 136) or neutral lipids (*n* = 97). The major phospholipid subclasses comprised Phosphatidylcholines (PC,*n* = 45), Phosphatidylethanolamines (PE, *n* = 43), Plasmenylphosphatidylethanolamines (PPE, *n* = 29) and Plasmenylphosphatidylcholines (PPC, *n* = 8). The neutral lipids included subclasses of Triacylglycerols (TG, *n* = 70), Cholesterol esters (CE, *n* = 19) and Diacylglycerols (DG, n = 7). Inherent differences in the lipidomic profiles were visualized using descriptive Principal Component Analysis (PCA) that showed a clear separation between different cancer cell lines (Fig. 1a). As expected, lipidomic profiles of cell lines from similar tissue of origin were more comparable to one another. To further elucidate the individual compounds contributing predominantly to the variance observed in lipidomic data from the selected cell lines a partial least-square-discrimination analysis (PLS-DA) model was constructed. The PLS-DA score plots also showed clear separation between different cell lines (Fig. 1a). Variable importance in the projection (VIP) values were applied (VIP values > 2.0) to identify 15 most important lipid molecules which mainly contributed in differentiating the lipidomic profiles of the cell lines (Fig. 1b). Ten of these lipid molecules were phospholipids, whereas five were triglycerides.

**Table 1:** Identified lipid classes and numbers of detected lipid molecules

Lipid Type	Lipid Class	Number of molecules
Phospholipids	Phosphatidylcholines (PC)	45
	Lysophosphatidylcholines (lysoPC)	2
	Lysophosphatidylethanolamines (lysoPE)	4
	Phosphatidylglycerols (PG)	1
	Plasmenylphosphatidylethanolamines (PPE)	29
	Plasmenylphosphatidylcholines (PPC)	8
	Phosphatidylethanolamines (PE)	43
	Bis(monoacylglycero)phosphate (BMP)	4
Neutral Lipids	Cholesterol ester (CE)	19
	Monoacyglycerol (MG)	1
	Diacylglycerol (DG)	7
	Triacylglycerol (TG)	70
Sphingomyelines	Sphingomyelines (SM)	4
Acylcarnitine	Acylcarnitine	5
Lanosteryl oleate	Lanosteryl oleate	1
Lanosteryl palmitoleate	Lanosteryl palmitoleate	1
Total		244

### Effect of metabolic stress on cell proliferation in cancer cells

**Fig. 2:**
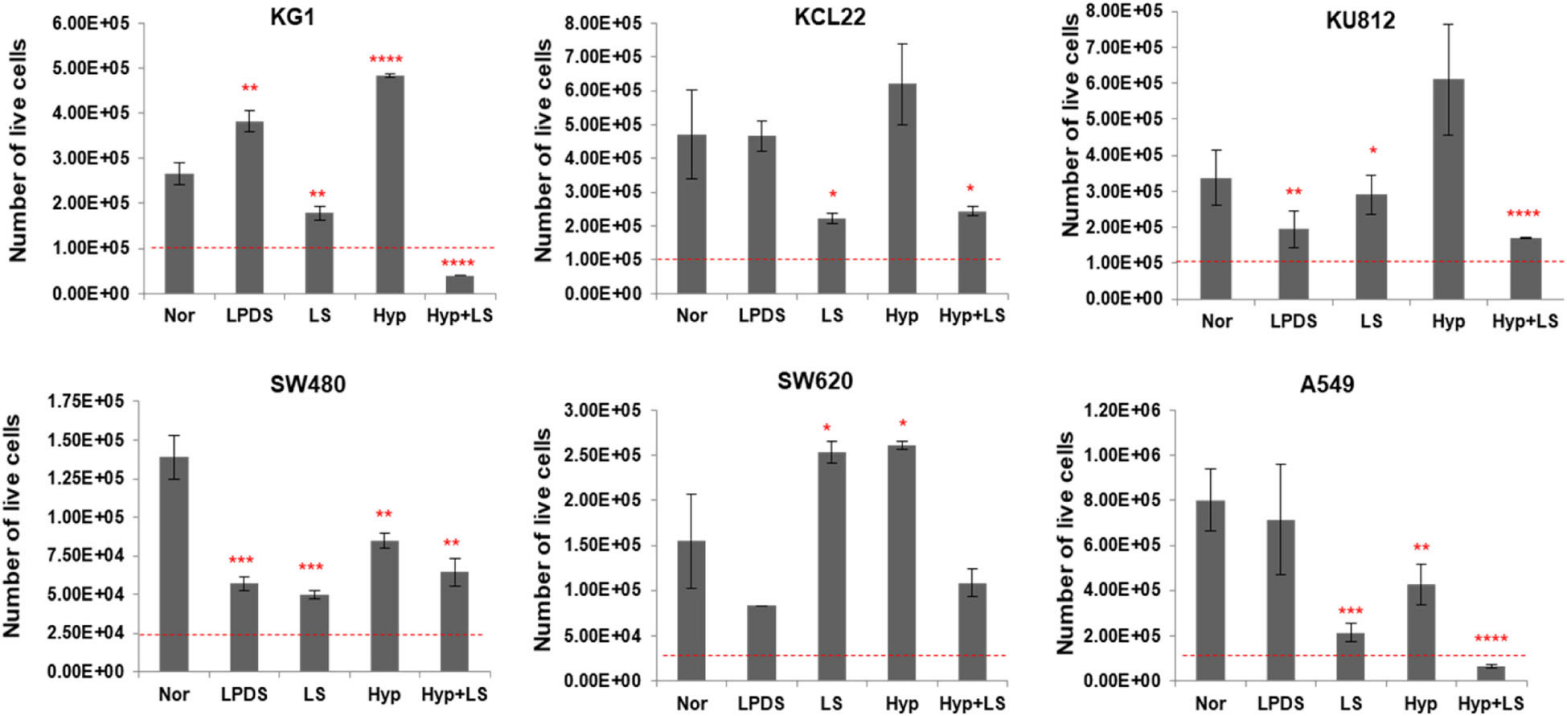
Effect of metabolic stress on cell proliferation in different cancer cell lines. KCL22 (Leukemia), KG1 (Leukemia), KU812 (Leukemia), SW480 (Colon cancer), SW620 (Colon cancer) and A549 (Lung Cancer) cells were cultivated under lipoprotein deficient medium (LPDS serum), low-serum (LS) medium (2% serum), hypoxia (Hyp, 2% O_2_), or hypoxia in combination with LS medium (Hyp + LS). The number of live cells was counted using a trypan blue dye exclusion method, after 48 h of culturing. The *dashed line* indicates the initial seeding density of cells.*Significantly different (*p ≤ 0,05; **p ≤ 0,01; ***p ≤ 0,001), n.s not significant (*p* > 0,05)

We have previously shown that cancer cells cultivated under low-lipid conditions display differential growth patterns [31]. Here, we studied the effect of different metabolically-stressed culture conditions on cell proliferation in various cancer cell lines. To induce metabolic stress, cells were cultivated for 48 h under following conditions: lipoprotein deficient medium (LPDS serum), low-serum medium (2% serum), hypoxia (2% O_2_), or hypoxia in combination with low-serum medium. Figure 2 shows number of live cancer cells cultivated under different conditions. We observed that metabolic stress significantly impacted proliferation rates of most cell lines (Fig. 2). KU812, SW480 and SW620 showed decreased proliferation rates when cultivated in media containing lipoprotein-deficient serum (LPDS). All cell lines except SW620 showed reduced proliferation rates under low-serum environment. Hypoxic conditions induced decreased proliferation rates in A549 and SW480. When cultivated under hypoxia in combination with low-serum medium all cell lines except SW620 displayed reduced proliferation rates.

### Effect of metabolic stress on lipidomic profile of cancer cells

**Fig. 3:**
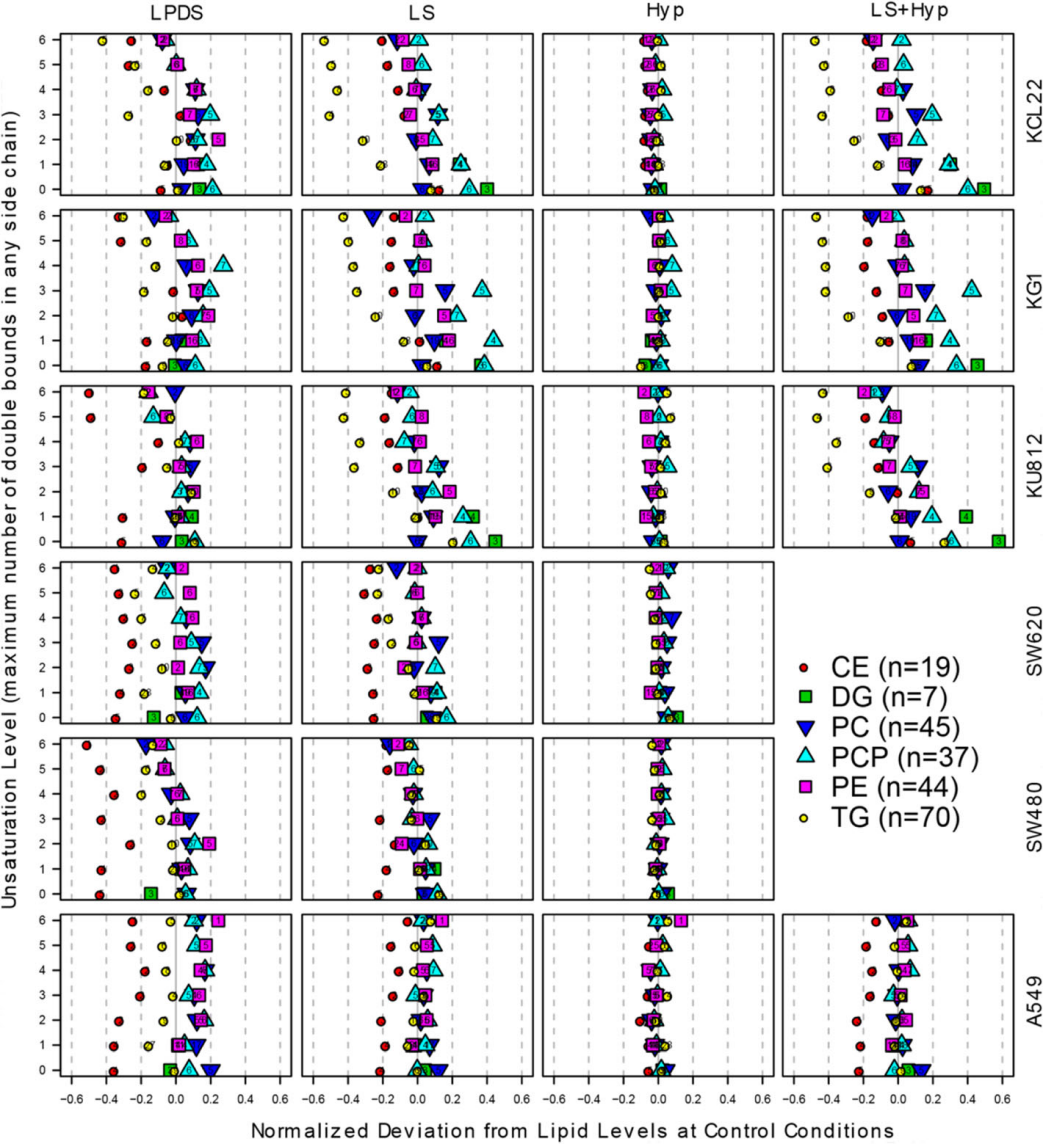
Effect of metabolic stress on lipidomic profiles of cancer cells: Each column shows changes in cellular levels of the six major lipid classes –including CEs, DGs, PCs PPCs, PEs and TG– under a specific stress condition relative to control for KCL22 (Leukemia), KG1 (Leukemia), KU812 (Leukemia), SW480 (Colon cancer), SW620 (Colon cancer) and A549 (Lung Cancer) cells. For each lipid class the peak intensities of the subspecies containing similar number of double bonds in their fatty acyl chains (chain containing highest number of double bonds) were summed up and the data were log2 transformed and median normalized

Next, we studied the effects of metabolic stress on lipidomic profiles of cancer cells. Previous studies have already shown various stress-related effects on individual lipid-moieties. Our broad lipidomic assay allowed us to assess the impact of metabolic stress on robust averages of broader lipid-classes, providing a more holistic overview of cancer cell lipidomic profiles. To this end, we only included lipid sub-groups with *n* > 6 to ensure statistical robustness (CEs, DGs, PCs PPCs, PEs and TGs). To further increase robustness of the analysis, for each of the analyzed lipid classes all subspecies containing similar saturation status were averaged. Figure 3 summarizes the data on lipidomic profiles of all cell lines cultivated under different metabolically-stressed conditions. To account for differences in cell number and hence total amount used in analysis we expressed each lipid peak intensity relative to the median peak intensity of the sample. The data is expressed relative to the baseline level (without stress) of the respective cell line. In each panel the data-points spreading away from the median-plane display differences from the normal conditions, color coded for lipid classes and stratified according to saturation index of their fatty-acyl side chains (expressed as the largest number of double bonds in any acyl side chain) on the second dimension (see Additional file 1: Supplementary Text Box 1 for explanation). We observed that cells cultivated under LPDS and LS containing media show multiple aberrations in their lipidomic profiles (Fig. 3). Almost all cell lines showed significant decrease in cellular levels of CE under lipoprotein deficient conditions. Under LS medium all leukemic cells displayed decreased levels of CE of polyunsaturated fatty acyl chains. However, the cell lines derived from solid tumors displayed overall decrease in CE under serum-deprivation. Colorimetric quantification of CE content also displayed significant reduction in total CE levels under LS and LPDS conditions (Additional file 2: Figure S1).

Most striking alterations include changes in TG profiles of leukemia cell lines (KG1, KCL22 and KU812) cultivated under LS medium. In leukemia cells under serum-deprived conditions the cellular level of TG subspecies decreased with increasing number of double bonds in their fatty acyl chains. A similar but slightly less pronounced effect was observed in SW620 cells. In other words, LS medium induced decreased levels of polyunsaturated fatty acid-enriched TGs in selected cancer cell lines. We also checked the impact of metabolic stress on total TG content in cancer cells. It was observed that all the leukemia cell lines and A549 cells display significant reductions in total TG content under LS and Hyp + LS conditions (Additional file 3: Figure S2). Levels of all DG sub-species were increased in leukemia cell lines under LS conditions (Fig. 3). Moreover, the levels of highly saturated PPCs were also significantly increased under LS condition particularly in KG1, KCL22 and KU812 cells. Cellular levels of various PC subspecies did not display marked changes or specific trends under metabolic stress. Surprisingly, we did not observe any robust changes in lipidomic profiles of cancer cells cultivated under hypoxia (Fig. 3). These results are strikingly clear for all of the detected lipid classes.

**Fig. 4:**
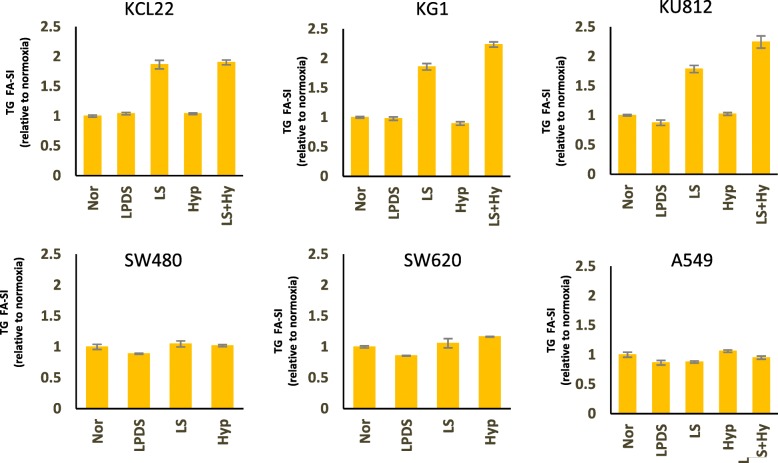
Fatty acid saturation indices (FA-SI) of triglycerides (TGs) in KCL22 (Leukemia), KG1 (Leukemia), KU812 (Leukemia), SW480 (Colon cancer), SW620 (Colon cancer) and A549 (Lung Cancer) cell lines under Nor, LPDS, LS, Hyp or Hyp + LS conditions

**Fig. 5:**
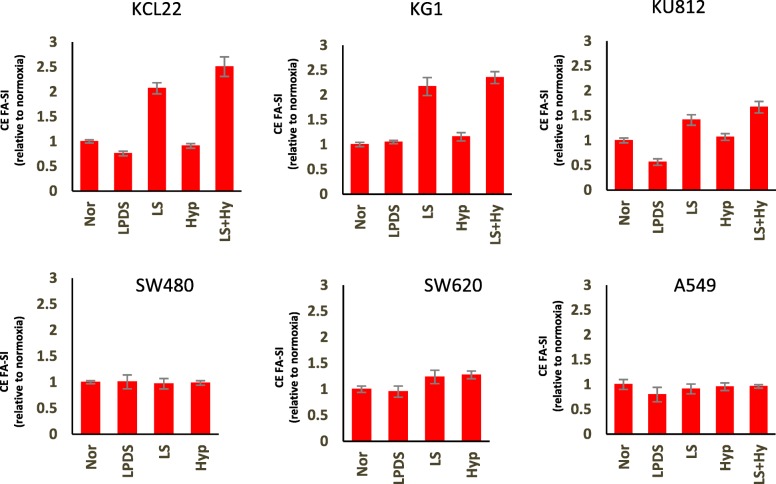
Fatty acid saturation indices (FA-SI) of cholesterol esters (CEs) in KCL22 (Leukemia), KG1 (Leukemia), KU812 (Leukemia), SW480 (Colon cancer), SW620 (Colon cancer) and A549 (Lung Cancer) cell lines under Nor, LPDS, LS, Hyp or Hyp + LS conditions

Our analyses revealed that LS conditions induce most pronounced changes in TG profiles of leukemia cells. Recently, *Ackerman* et al [27] published that LS or Hyp + LS condition induces a loss of TGs harboring unsaturated FAs and a shift toward increased TG saturation in renal cancer cells. In this paper, TGs were stratified according to the number of attached SFAs (i.e. 0 SFA, 1 SFA or ≥ 2 SFA). To further elucidate our findings we also stratified TGs on these basis. We observed that under low-serum (LS) condition the composition of TGs was significantly altered and the proportion of TGs with ≥2 SFA was markedly increased in all leukemia cell lines (Additional file 4: Figure S3). Under hypoxic condition the FA composition of TGs did not significantly alter in comparison to the normal condition. However, when hypoxia was induced in combination with LS condition (Hyp + LS) the proportion of TGs with ≥2 SFA was drastically increased together with a loss of TGs harboring unsaturated FAs. This effect was more pronounced than that observed under LS condition only. SW480, SW620 and A549 on the other hand, did not display such drastic effects under LS condition. This stratification system showed slight increase in TGs harboring ≥2 SFA in hypoxic SW480, SW620 and A549 cells. However, when TG saturation-indices (SI) were compared no significant changes were observed under hypoxia in any of the selected cell lines (Fig. 4). In line with our other analyses, the SI of TGs were significantly increased under LS and Hyp + LS condition in all leukemia cell lines (Fig. 4). LS and Hyp + LS condition also induced significant increase in SIs of DGs (Additional file 5: Figure S4) and CEs (Fig. 5) in leukemia cells. No other significant changes in SIs were observed for other lipid classes (Additional file 6: Figure S5 and Additional file 7: Figure S6).

Most of the previous studies have separately compared data for each lipid subspecies, whereas here we compared subgroups classified according to the number of double bonds in their fatty acyl chains. When individual lipid molecules were compared, indeed certain changes in the cellular levels of various lipid subspecies could be observed. Additional file 8: Table S1 compares levels of various lipid subspecies in A549 cells cultivated under normoxia vs. hypoxia. Here, we also observed significant alterations in the levels of various lipid subspecies. Some of these changes were in line with published data [25, 26].

## Discussion

The major goal of the presented work was to study the impact of nutrient and oxygen-deprivation on lipidomic profiles of cancer cells. The most profound alterations were observed in TG compositions, particularly of leukemia cells under LS environments, including a loss of TGs harboring unsaturated FAs and a shift toward increased TG saturation. These results are in line with the recently published work by *Ackerman* et al [27] that reported increase in TG saturation index under Hyp + LS or LS conditions in renal cancer cells. It has been previously shown that cancer cells preferentially utilize exogenous unsaturated fatty acids available from the culture media or tumor microenvironment, until they become limiting [32].We hypothesize that under LS environment there is a toxic buildup of saturated lipids due to limited supply of environmental PUFAs. To overcome that the unsaturated fatty acids bound to the stored TGs are released and the cellular lipid homeostasis is maintained. This escape mechanism of releasing TG-bound unsaturated fatty acids to counteract toxic buildup of saturated lipids has also been observed in cancer cells in which de novo desaturation pathway is inhibited [27]. However, LS-mediated induction of this effect is not observed in all cell lines, for instance, SW480 and A549 do not display any systematic changes in their TG profiles under LS conditions. SW480 cells display highest baseline TG saturation index among the selected cell line (Additional file 9: Figure S7), in other words, the baseline TG-deposits in SW480 may not have higher amounts of bound PUFAs to counteract toxic buildup of saturated lipids under serum-deprivation. Hence, these cells may use other mechanisms to overcome the unsaturated fatty acids deficiency. For instance, *Roongta* et al. [32] have shown that A549 cells along with several cancer cell lines enables endogenous biogenesis of unsaturated fatty acids under LS condition.

We observed that in addition to severe modification in their TG profiles the cancer cells also display significant reductions in cellular levels of all CE subspecies. TGs along with CEs are the major constituents of lipid droplets (LD). Previous works have shown that serum-deprivation induces LD-depletion in different cancer cell types [33–35]. It has been previously reported that under restricted cholesterol-rich low-density lipoprotein (LDL) supply cancer cells mobilize CEs [36]. Hence, it is possible that under LS conditions cancer cells rely on previously stored LD content for the release of free fatty acids that subsequently caused decrease in CE and TG levels.

The most striking observation of this study was that the lipidomic profiles of cancer cells remained robust under hypoxia. This effect was consistent throughout the selected cell line panel and for all the analyzed lipid sub-groups. This effect was particularly interesting because we observed changes in proliferation capacity, expression of lipid synthesis/degradation markers (Additional file 10: Figure S8) and expression of hypoxia markers (data not shown) in hypoxic cancer cells. Despite all these changes cancer cells maintained homeostasis for all lipid classes. Also, if stresses are combined (LS + Hyp) the observed pattern is similar to LS conditions and not altered by hypoxia. Few previous studies reported that hypoxia induces changes in phospholipid/triglyceride profiles of cancer cells [25, 26]. Previous works compared the levels of individual lipid moieties and observed significant changes in multiple TG and PC subspecies. We also observed changes in cellular levels of various lipid subspecies when compared individually (Additional file 8: Table S1). However, our broad lipidomic assay allowed us to focus on robust averages from larger lipid-classes classified on the basis of saturation-density of their fatty acyl chains. These analyses provide a more holistic view of saturation status of various lipid classes. A previous study by Yu et al. [25]–that reported clear cut changes in PC profiles of Hela cells under hypoxia– also noted that changes in PC profiles are only evident when individual PC species are analyzed. However, when the relative abundance for PL species with acyl chains containing ≥3 double bonds were compared with that containing <3double bonds no significant difference was observed between cells under normoxia and hypoxia. Literature survey reveales differences in cell culture methods among previous studies that may also lead to contradictory evidence. For instance; cells were serum-starved prior to hypoxia-induction [25], hypoxia was applied in combination with nutrient-deprivation [26] or full serum media was supplemented with exogenous lipids [37]. Here, we also observed that the lipidomic profiles of the selected cancer cell lines cultivated under low-serum environment were similar to that cultivated under hypoxia+low-serum environment. Hence, one can speculate the altered lipidomic profiles are mainly regulated by nutrient-availability and not by hypoxia. Further studies are required to understand the molecular mechanisms regulating lipidomic profiles of cancer cells under metabolic stress.

## Conclusions

The presented work aimed to determine the impact of metabolic stress on lipidomic profiles in biologically diverse cancer cells. Our data showed that nutrient deprivation leads to systematic changes in lipidomic profiles -particularly neutral lipid composition- of cancer cells. Unexpectedly and in contrast to previously published data, we observe that low oxygen conditions do not systematically affect cancer cell lipid composition despite concurrent changes in proliferation rates and metabolic gene expression. Most of the previous studies have separately compared data for each lipid subspecies, whereas here we compared subgroups classified according to the number of double bonds in their fatty acyl chains. We also observed certain changes in the cellular levels of various lipid subspecies when individual lipid molecules were compared. We conclude that although the levels of individual lipid moieties alter under hypoxia, the robust averages of broader lipid class remain unchanged.

## Additional files

Additional file 1: Supplementary Text Box 1. (PPTX 1809 kb)

Additional file 2: **Figure S1.** Fold-changes in total cholesterol ester (CE) content in KCL22 (Leukemia), KG1 (Leukemia), KU812 (Leukemia), SW480 (Colon cancer), SW620 (Colon cancer) and A549 (Lung Cancer) cell lines under Nor, LPDS, LS, Hyp or Hyp+LS conditions. (PPTX 849 kb)

Additional file 3: **Figure S2.** Fold-changes in triglyceride-content in KCL22 (Leukemia), KG1 (Leukemia), KU812 (Leukemia), SW480 (Colon cancer), SW620 (Colon cancer) and A549 (Lung Cancer) cell lines under Nor, LPDS, LS, Hyp or Hyp+LS conditions. (PPTX 849 kb)

Additional file 4: **Figure S3.** Proportion of TGs containing 0, 1 and ≥2 SFA in (a) KCL22 (Leukemia) (b) KG1 (Leukemia) (c) KU812 (Leukemia) (d) SW480 (Colon cancer) (e) SW620 (Colon cancer) (f) A549 (Lung Cancer) cell lines under Nor, LPDS, LS, Hyp or Hyp+LS conditions. (PPTX 204 kb)

Additional file 5: **Figure S4.** Fatty acid saturation indices (FA-SI) of diglycerides (DGs) in (a) KCL22 (Leukemia) (b) KG1 (Leukemia) (c) KU812 (Leukemia) (d) SW480 (Colon cancer) (e) SW620 (Colon cancer) (f) A549 (Lung Cancer) cell lines under Nor, LPDS, LS, Hyp or Hyp+LS conditions. (PPTX 71 kb)

Additional file 6: **Figure S5.** Fatty acid saturation indices (FA-SI) of phosphatidylcholine (PC) in (a) KCL22 (Leukemia) (b) KG1 (Leukemia) (c) KU812 (Leukemia) (d) SW480 (Colon cancer) (e) SW620 (Colon cancer) (f) A549 (Lung Cancer) cell lines under Nor, LPDS, LS, Hyp or Hyp+LS conditions. (PPTX 71 kb)

Additional file 7: **Figure S6.** Fatty acid saturation indices (FA-SI) of phosphatidylethanolamine (PE) in (a) KCL22 (Leukemia) (b) KG1 (Leukemia) (c) KU812 (Leukemia) (d) SW480 (Colon cancer) (e) SW620 (Colon cancer) (f) A549 (Lung Cancer) under Nor, LPDS, LS, Hyp or Hyp+LS conditions. (PPTX 71 kb)

Additional file 8: **Table S1.** Changes in abundance of individual lipid moieties under hypoxia in A549 cells. The data were analyzed by the univariate ANOVA analysis for repeated measures (significant *p-value < 0.001). *P*-values for the lipid species significantly altered are indicated in bold (red font). The left- most column indicates the lipid moieties reported to be significantly altered (√) under hypoxic condition by previous works (in different cell line models). (DOCX 41 kb)

Additional file 9: **Figure S7.** Baseline saturation indices of TGs in KCL22 (Leukemia), KG1 (Leukemia), KU812 (Leukemia), SW480 (Colon cancer), SW620 (Colon cancer), A549 (Lung Cancer). (PPTX 99 kb)

Additional file 10: **Figure S8.** Effect of metabolic stress on expression of selected genes from de novo lipid synthesis or lipid uptake/degradation pathways in different cancer cell lines. Box plots showing log2 transformed and median normalized values for (a) FASN (b) HMGCR (c) MGLL expression levels in KCL22 (Leukemia), KG1 (Leukemia), KU812 (Leukemia), SW480 (Colon cancer), SW620 (Colon cancer) and A549 (Lung Cancer). (d) LPL expression level in KU812 (Leukemia) and SW480 (Colon cancer) cells. (e) CD36 expression level in KU812 (Leukemia) cells. Cells were cultivated (48 hours) under lipoprotein deficient medium (LPDS serum), low-serum (LS) medium (2% serum), hypoxia (2% O2), or hypoxia in combination with LS medium. The levels of the different transcripts were measured in 3 to 6 samples by qPCR. The results show the distribution of corresponding transcripts relative to GAPDH, with the box indicating the 25th–75th percentiles, with the median indicated. line. The whiskers show the range. Data were normalized to the median expression level of the given transcript under normal conditions for the respective cell line. (PPTX 218 kb)

## Data Availability

The datasets supporting the conclusions of this article are included within the article and supplementary files.

## Abbreviations

CE: Cholesterol esters
DG: Diacylglycerols
FASN: Fatty acid synthase
HMGCR: 3-hydroxy-3-methylglutaryl-CoA reductase
LPL: Lipoprotein lipase
MGLL: Monoacylglycerol lipase
PC: Phosphatidylcholines
PE: Phosphatidylethanolamines
PPC: Plasmenylphosphatidylcholines
PPE: Plasmenylphosphatidylethanolamines
TG: Triacylglycerols
