# Supplementary material for: Cancer cell lipid class homeostasis is altered under nutrient-deprivation but stable under hypoxia
Source: BMC Cancer. 2019 May 28;19:501. doi: 10.1186/s12885-019-5733-y (PMC6537432; doi:10.1186/s12885-019-5733-y)
Supplement: Supplementary file 1 — Supplementary Text Box 1. (PPTX 1809 kb) [file 12885_2019_5733_MOESM1_ESM.pptx]

## Slide 1
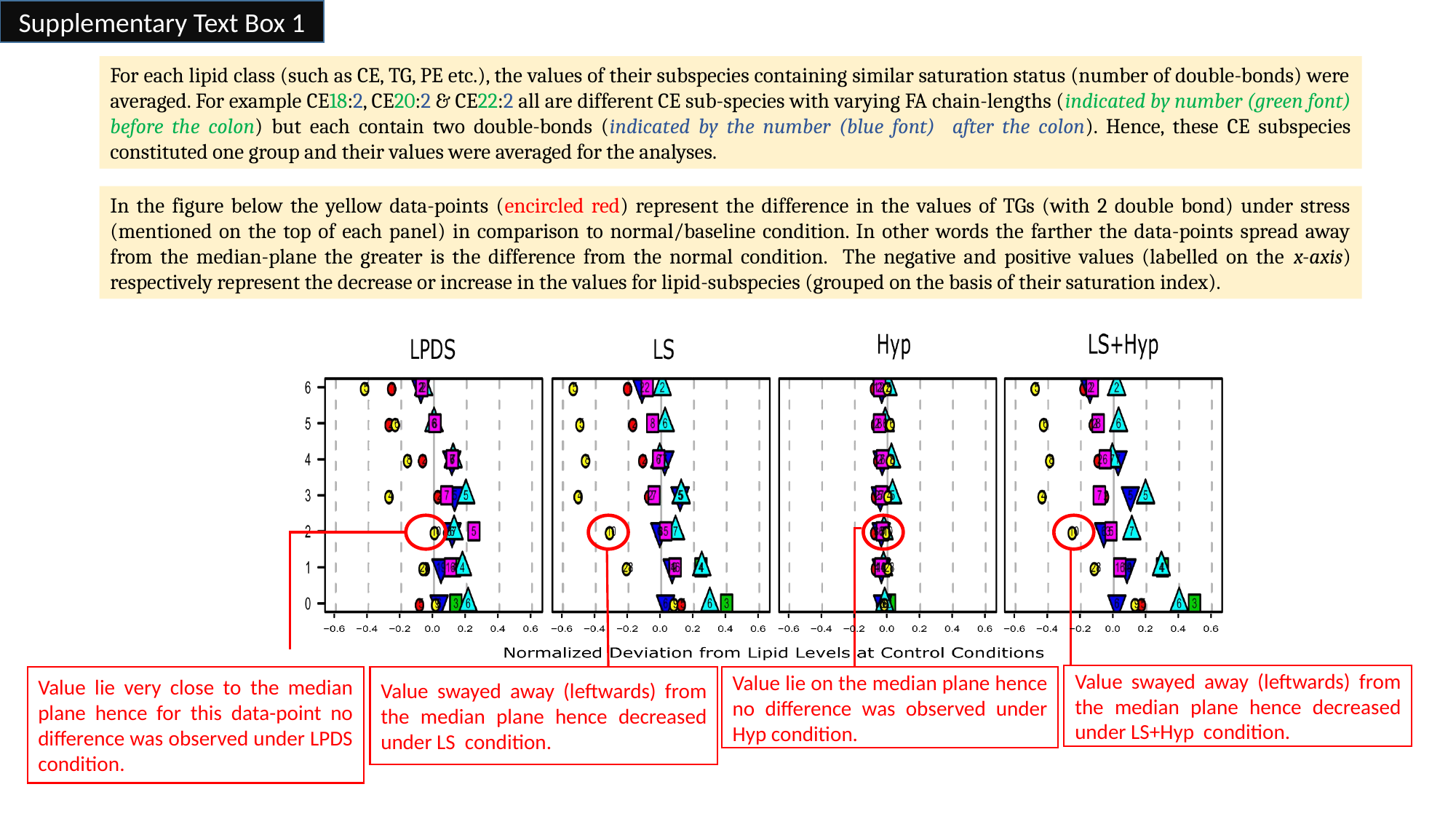

Supplementary Text Box 1
For each lipid class (such as CE, TG, PE etc.), the values of their subspecies containing similar saturation status (number of double-bonds) were averaged. For example CE18:2, CE20:2 & CE22:2 all are different CE sub-species with varying FA chain-lengths (indicated by number (green font) before the colon) but each contain two double-bonds (indicated by the number (blue font) after the colon). Hence, these CE subspecies constituted one group and their values were averaged for the analyses.
In the figure below the yellow data-points (encircled red) represent the difference in the values of TGs (with 2 double bond) under stress (mentioned on the top of each panel) in comparison to normal/baseline condition. In other words the farther the data-points spread away from the median-plane the greater is the difference from the normal condition. The negative and positive values (labelled on the x-axis) respectively represent the decrease or increase in the values for lipid-subspecies (grouped on the basis of their saturation index).
Value swayed away (leftwards) from the median plane hence decreased under LS+Hyp condition.
Value lie very close to the median plane hence for this data-point no difference was observed under LPDS condition.
Value lie on the median plane hence no difference was observed under Hyp condition.
Value swayed away (leftwards) from the median plane hence decreased under LS condition.
