# Supplementary material for: Cancer cell lipid class homeostasis is altered under nutrient-deprivation but stable under hypoxia
Source: BMC Cancer. 2019 May 28;19:501. doi: 10.1186/s12885-019-5733-y (PMC6537432; doi:10.1186/s12885-019-5733-y)
Supplement: Supplementary file 2 — Figure S1. Fold-changes in total cholesterol ester (CE) content in KCL22 (Leukemia), KG1 (Leukemia), KU812 (Leukemia), SW480 (Colon cancer), SW620 (Colon cancer) and A549 (Lung Cancer) cell lines under Nor, LPDS, LS, Hyp or Hyp+LS conditions. (PPTX 849 kb) [file 12885_2019_5733_MOESM2_ESM.pptx]

## Slide 1
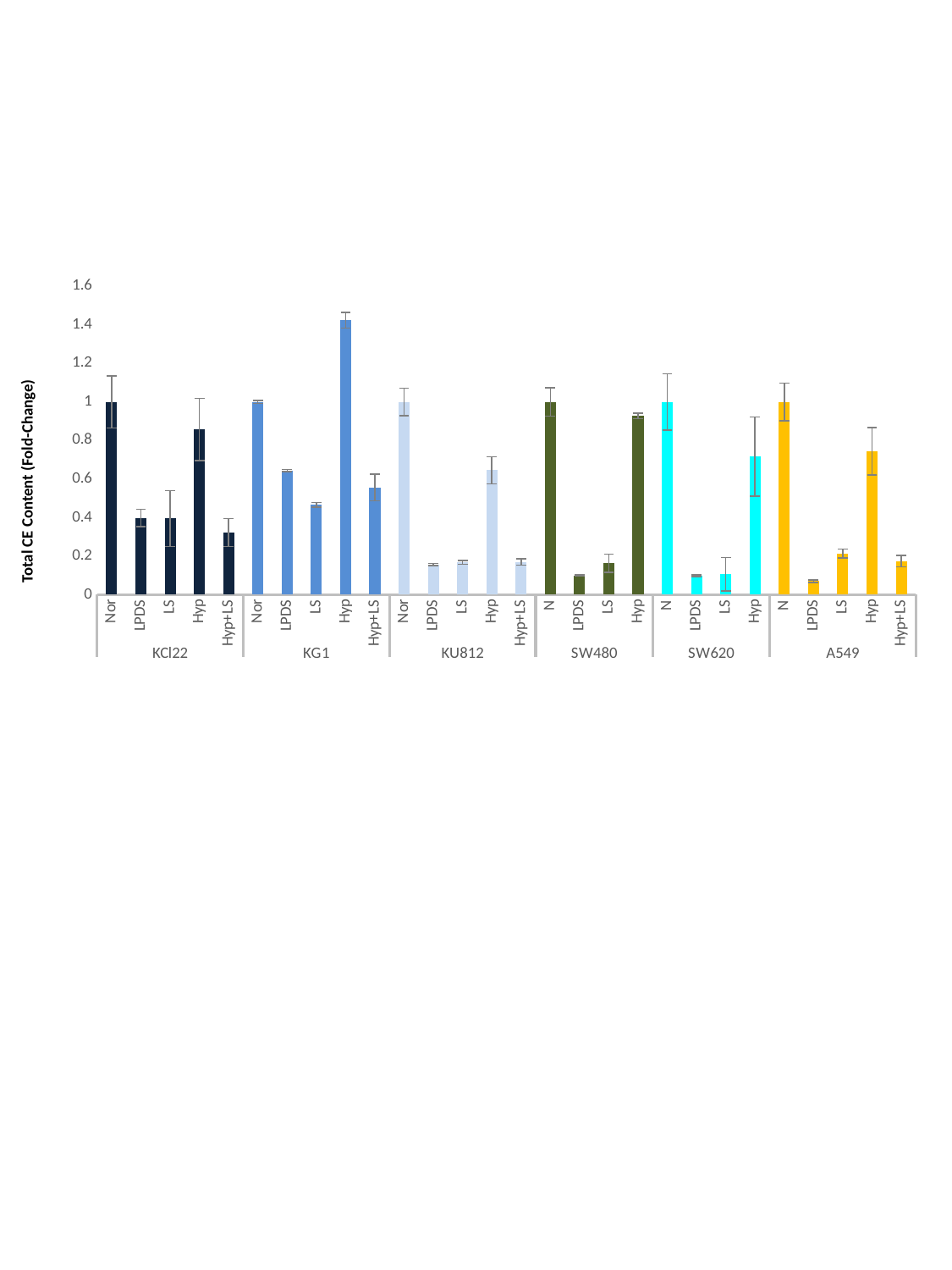

### Chart
| Category | |
|---|---|
| Nor | 1.0 |
| LPDS | 0.39760293345501396 |
| LS | 0.3950631946093937 |
| Hyp | 0.8567948269180289 |
| Hyp+LS | 0.322350717580728 |
| Nor | 1.0 |
| LPDS | 0.6448317583814501 |
| LS | 0.46657987839431503 |
| Hyp | 1.4245294288821158 |
| Hyp+LS | 0.5564700657038274 |
| Nor | 1.0 |
| LPDS | 0.1567777612559967 |
| LS | 0.16784246860608823 |
| Hyp | 0.6456402114063561 |
| Hyp+LS | 0.1693321038328278 |
| N | 1.0 |
| LPDS | 0.10020547807835721 |
| LS | 0.16316330318371525 |
| Hyp | 0.9274842398025989 |
| N | 1.0 |
| LPDS | 0.09825832650804697 |
| LS | 0.10579854182439952 |
| Hyp | 0.7169735427766767 |
| N | 1.0 |
| LPDS | 0.07013010830832496 |
| LS | 0.21303487804598464 |
| Hyp | 0.7437676835275121 |
| Hyp+LS | 0.17402967239428765 |Total CE Content (Fold-Change)
