# Supplementary material for: Cancer cell lipid class homeostasis is altered under nutrient-deprivation but stable under hypoxia
Source: BMC Cancer. 2019 May 28;19:501. doi: 10.1186/s12885-019-5733-y (PMC6537432; doi:10.1186/s12885-019-5733-y)
Supplement: Supplementary file 3 — Figure S2. Fold-changes in triglyceride-content in KCL22 (Leukemia), KG1 (Leukemia), KU812 (Leukemia), SW480 (Colon cancer), SW620 (Colon cancer) and A549 (Lung Cancer) cell lines under Nor, LPDS, LS, Hyp or Hyp+LS conditions. (PPTX 849 kb) [file 12885_2019_5733_MOESM3_ESM.pptx]

## Slide 1
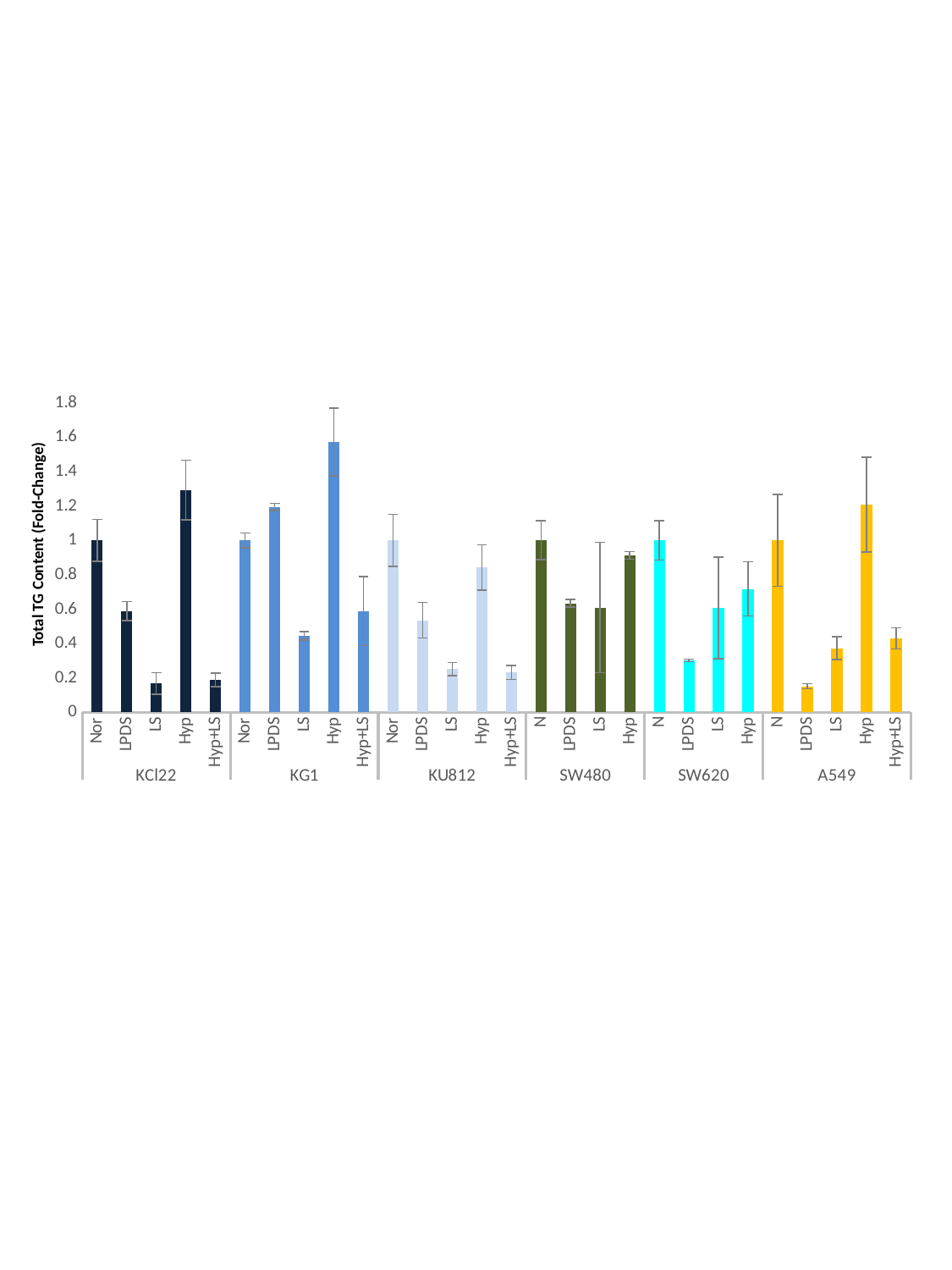

### Chart
| Category | |
|---|---|
| Nor | 1.0 |
| LPDS | 0.5883450205003091 |
| LS | 0.16726613658025366 |
| Hyp | 1.2923726183552555 |
| Hyp+LS | 0.1894265137159439 |
| Nor | 1.0 |
| LPDS | 1.193942790444512 |
| LS | 0.44304630650816734 |
| Hyp | 1.5717360204159074 |
| Hyp+LS | 0.5887735174921468 |
| Nor | 1.0 |
| LPDS | 0.535123754139136 |
| LS | 0.2503578302402489 |
| Hyp | 0.842658143112225 |
| Hyp+LS | 0.23099536910922902 |
| N | 1.0 |
| LPDS | 0.6329852609212445 |
| LS | 0.6086867019628102 |
| Hyp | 0.9122817010702492 |
| N | 1.0 |
| LPDS | 0.30085237356752026 |
| LS | 0.605900265361842 |
| Hyp | 0.7176061367511894 |
| N | 1.0 |
| LPDS | 0.1506836338276991 |
| LS | 0.3727616168557703 |
| Hyp | 1.2073186683433659 |
| Hyp+LS | 0.42914511946215994 |Total TG Content (Fold-Change)
