# Supplementary material for: Cancer cell lipid class homeostasis is altered under nutrient-deprivation but stable under hypoxia
Source: BMC Cancer. 2019 May 28;19:501. doi: 10.1186/s12885-019-5733-y (PMC6537432; doi:10.1186/s12885-019-5733-y)
Supplement: Supplementary file 4 — Figure S3. Proportion of TGs containing 0, 1 and ≥2 SFA in (a) KCL22 (Leukemia) (b) KG1 (Leukemia) (c) KU812 (Leukemia) (d) SW480 (Colon cancer) (e) SW620 (Colon cancer) (f) A549 (Lung Cancer) cell lines under Nor, LPDS, LS, Hyp or Hyp+LS conditions. (PPTX 204 kb) [file 12885_2019_5733_MOESM4_ESM.pptx]

## Slide 1
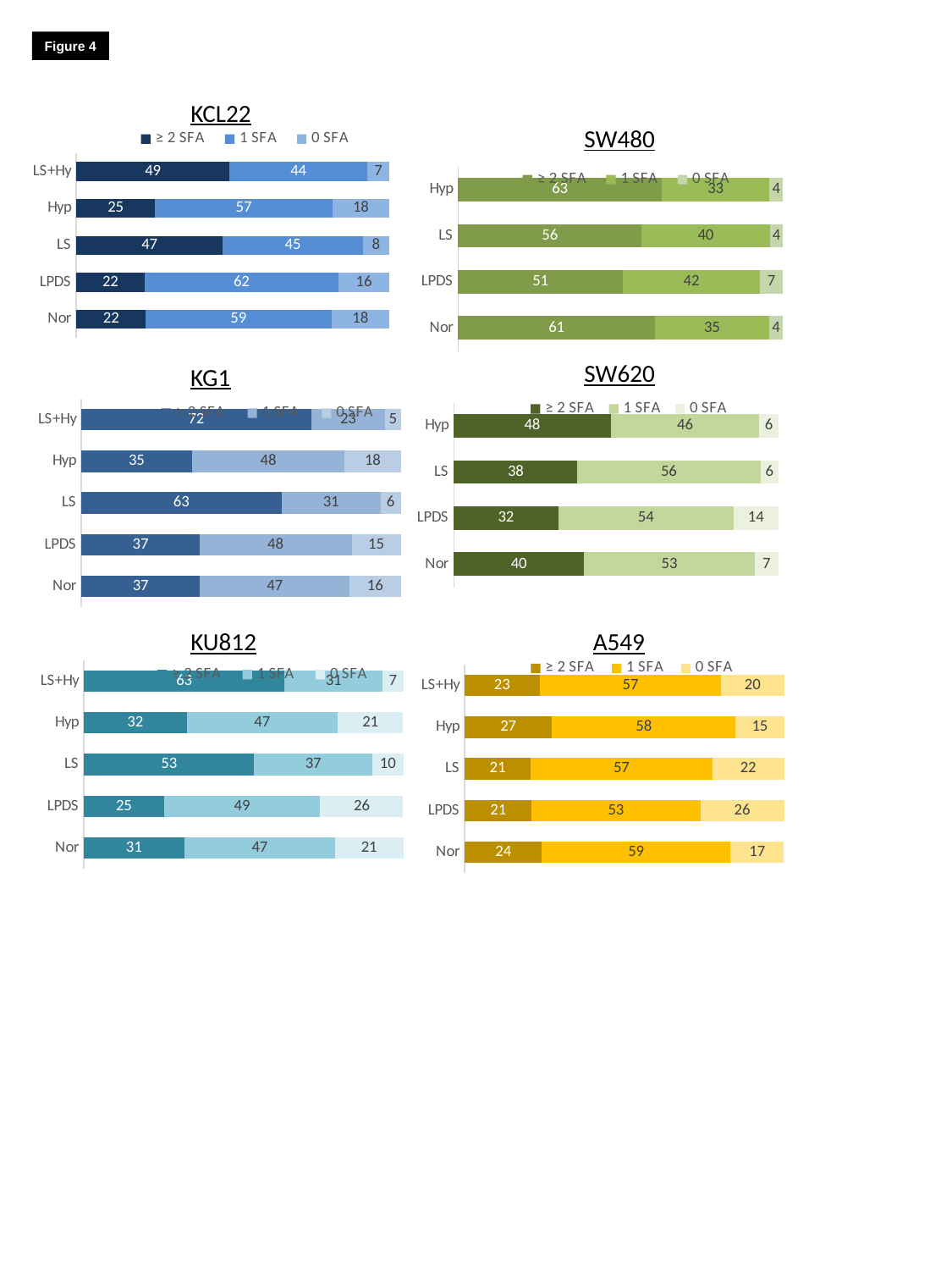

Figure 4
### Chart
| Category | ≥ 2 SFA | 1 SFA | 0 SFA |
|---|---|---|---|
| Nor | 22.16529082246053 | 59.47536722372652 | 18.359341953812898 |
| LPDS | 21.82461966901016 | 62.05960087325502 | 16.115779457734792 |
| LS | 46.76049295523876 | 44.906791933243895 | 8.332715111517324 |
| Hyp | 25.09852273158437 | 56.95435976546644 | 17.94711750294908 |
| LS+Hy | 48.97704430810646 | 44.14363291622213 | 6.879322775671391 |KCL22
SW480
### Chart
| Category | ≥ 2 SFA | 1 SFA | 0 SFA |
|---|---|---|---|
| Nor | 60.61838558414881 | 35.08441099737373 | 4.2972034184774595 |
| LPDS | 50.765437868556 | 42.13099237900369 | 7.103569752440351 |
| LS | 56.45089294690579 | 39.524914445190625 | 4.024192607903616 |
| Hyp | 62.647714235946346 | 33.173761314697245 | 4.178524449356403 |SW620
KG1
### Chart
| Category | ≥ 2 SFA | 1 SFA | 0 SFA |
|---|---|---|---|
| Nor | 37.10809760760998 | 46.954547874419035 | 15.93735451797096 |
| LPDS | 37.00961830380228 | 47.62783298152169 | 15.362548714676057 |
| LS | 62.7212041363336 | 30.985545912780886 | 6.293249950885508 |
| Hyp | 34.64895686271118 | 47.77676162967094 | 17.574281507617883 |
| LS+Hy | 71.95836079156996 | 23.138438883868286 | 4.9032003245617295 |
### Chart
| Category | ≥ 2 SFA | 1 SFA | 0 SFA |
|---|---|---|---|
| Nor | 40.07207389848386 | 52.58584464051486 | 7.3420814610013085 |
| LPDS | 32.18069837675602 | 53.92780182484772 | 13.891499798396257 |
| LS | 38.06845861374182 | 56.32408894489308 | 5.6074524413651075 |
| Hyp | 48.33701855420065 | 45.73284754753495 | 5.93013389826438 |A549
### Chart
| Category | ≥ 2 SFA | 1 SFA | 0 SFA |
|---|---|---|---|
| Nor | 31.368191729056704 | 47.339127103484664 | 21.292681167458635 |
| LPDS | 25.021668867305532 | 48.984832371494036 | 25.9934987612004 |
| LS | 53.28980614001745 | 37.0617355723006 | 9.648458287681951 |
| Hyp | 32.147653694231764 | 47.31526203557591 | 20.537084270192327 |
| LS+Hy | 62.85641526863625 | 30.61645304432459 | 6.5271316870391445 |KU812
### Chart
| Category | ≥ 2 SFA | 1 SFA | 0 SFA |
|---|---|---|---|
| Nor | 24.148354752241318 | 58.981663461383185 | 16.869981786375426 |
| LPDS | 20.96396993241582 | 52.844177156220795 | 26.191852911363366 |
| LS | 20.607408858122568 | 56.952929035757045 | 22.43966210612036 |
| Hyp | 27.261994347388207 | 57.60469292027312 | 15.133312732338663 |
| LS+Hy | 23.469904626828953 | 56.833871224510446 | 19.69622414866059 |
