# Supplementary material for: Cancer cell lipid class homeostasis is altered under nutrient-deprivation but stable under hypoxia
Source: BMC Cancer. 2019 May 28;19:501. doi: 10.1186/s12885-019-5733-y (PMC6537432; doi:10.1186/s12885-019-5733-y)
Supplement: Supplementary file 6 — Figure S5. Fatty acid saturation indices (FA-SI) of phosphatidylcholine (PC) in (a) KCL22 (Leukemia) (b) KG1 (Leukemia) (c) KU812 (Leukemia) (d) SW480 (Colon cancer) (e) SW620 (Colon cancer) (f) A549 (Lung Cancer) cell lines under Nor, LPDS, LS, Hyp or Hyp+LS conditions. (PPTX 71 kb) [file 12885_2019_5733_MOESM6_ESM.pptx]

## Slide 1
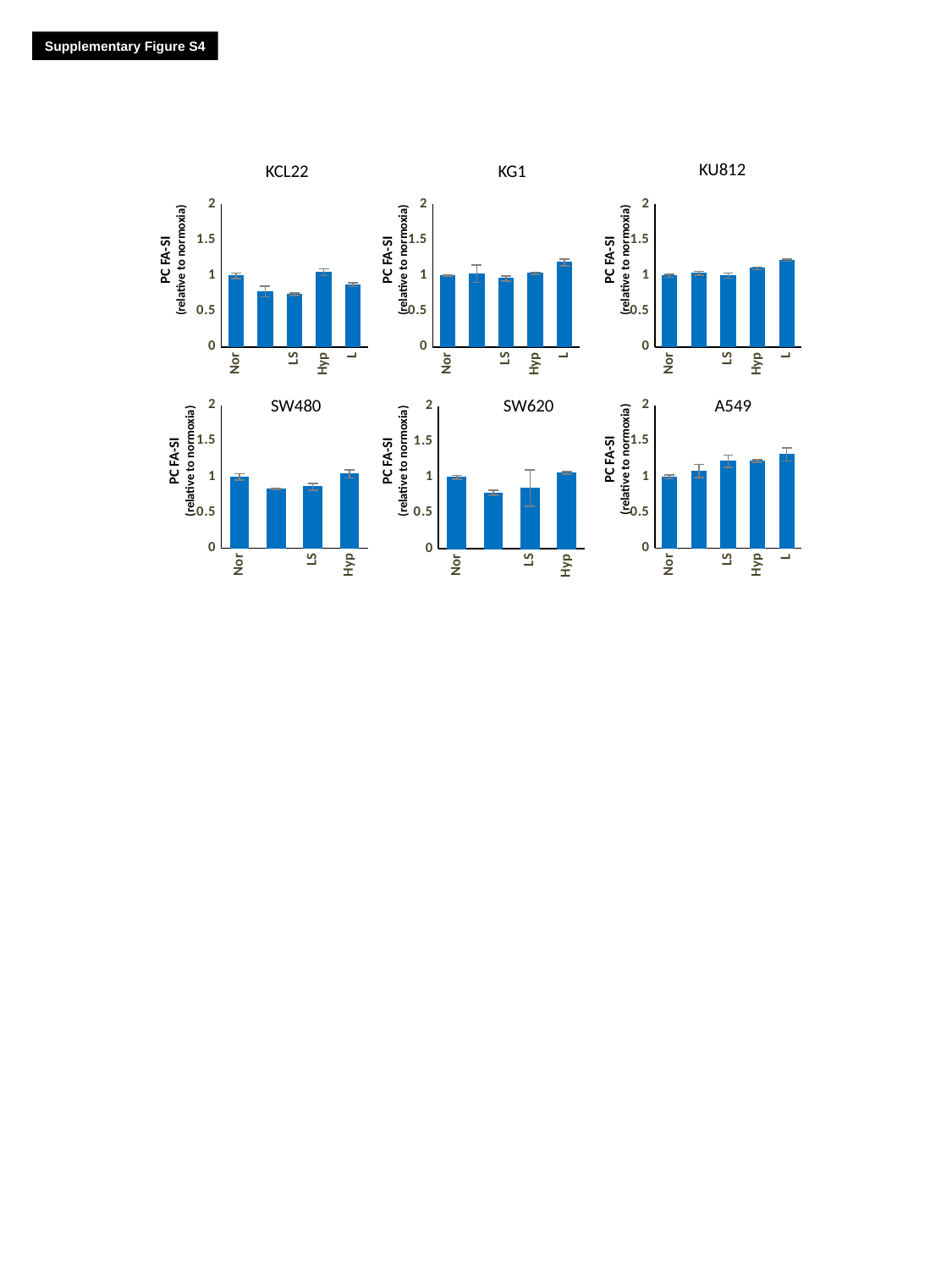

Supplementary Figure S4
KU812
KCL22
KG1
### Chart
| Category | Mean |
|---|---|
| Nor | 1.0 |
| LPDS | 0.7799924589537118 |
| LS | 0.7399469874835246 |
| Hyp | 1.0484975845363205 |
| LS+Hy | 0.8731600037705842 |
### Chart
| Category | Mean |
|---|---|
| Nor | 1.0 |
| LPDS | 1.027497474841876 |
| LS | 0.9600423248501938 |
| Hyp | 1.036153388398534 |
| LS+Hy | 1.1861215082911183 |
### Chart
| Category | Mean |
|---|---|
| Nor | 1.0 |
| LPDS | 1.0353774608479083 |
| LS | 1.0010397225603618 |
| Hyp | 1.104006275662866 |
| LS+Hy | 1.2169259845325913 |PC FA-SI
(relative to normoxia)
PC FA-SI
(relative to normoxia)
PC FA-SI
(relative to normoxia)
SW480
SW620
A549
### Chart
| Category | Mean |
|---|---|
| Nor | 1.0 |
| LPDS | 0.8263181130914834 |
| LS | 0.8583609579653606 |
| Hyp | 1.039818515638065 |
### Chart
| Category | Mean |
|---|---|
| Nor | 1.0000000000000002 |
| LPDS | 1.084008683911521 |
| LS | 1.2205749733260707 |
| Hyp | 1.223613206383786 |
| LS+Hy | 1.3141635188059322 |
### Chart
| Category | Mean |
|---|---|
| Nor | 1.0 |
| LPDS | 0.7828100754912397 |
| LS | 0.8488685601202036 |
| Hyp | 1.0651513481202093 |PC FA-SI
(relative to normoxia)
PC FA-SI
(relative to normoxia)
PC FA-SI
(relative to normoxia)
