# Supplementary material for: Cancer cell lipid class homeostasis is altered under nutrient-deprivation but stable under hypoxia
Source: BMC Cancer. 2019 May 28;19:501. doi: 10.1186/s12885-019-5733-y (PMC6537432; doi:10.1186/s12885-019-5733-y)
Supplement: Supplementary file 8 — Table S1. Changes in abundance of individual lipid moieties under hypoxia in A549 cells. The data were analyzed by the univariate ANOVA analysis for repeated measures (significant *p-value < 0.001). P-values for the lipid species significantly altered are indicated in bold (red font). The left- most column indicates the lipid moieties reported to be significantly altered (√) under hypoxic condition by previous works (in different cell line models). (DOCX 41 kb) [file 12885_2019_5733_MOESM8_ESM.docx]

| **Supplementary Table 1: Changes in abundance of individual lipid moieties under hypoxia in A549 cells.** The data were analyzed by the univariate ANOVA analysis for repeated measures (significant *p-value < 0.001). P-values for the lipid species significantly altered are indicated in bold (red font). The left-most column indicates the lipid moieties reported to be significantly altered (√) under hypoxic condition by previous works (in different cell line models). | | | | |
| --- | --- | --- | --- | --- |
| **Name** | **A549-Hyp/A549-N** | **p.value.origin** | **Significant difference** | **Significant differences in previous reports** |
| 14:0 CE | Down | 0.00201 | FALSE |  |
| 15:0 CE | Down | 0.82198 | FALSE |  |
| 16:0 CE | Down | **0.00071** | **TRUE** |  |
| 16:1 CE | Down | 0.00147 | FALSE |  |
| 17:0 CE | Down | 0.10975 | FALSE |  |
| 17:1 CE | Down | 0.28701 | FALSE |  |
| 18:0 CE | Down | 0.00287 | FALSE |  |
| 18:1 CE | Down | **0.00042** | **TRUE** |  |
| 18:2 CE | Down | **0.00009** | **TRUE** |  |
| 18:3 CE | Down | 0.80162 | FALSE |  |
| 20:1 CE | Down | **0.00001** | **TRUE** |  |
| 20:2 CE | Down | **0.00002** | **TRUE** |  |
| 20:3 CE | Down | **0.00096** | **TRUE** |  |
| 20:4 CE | Down | 0.50579 | FALSE |  |
| 20:5 CE | Down | 0.06494 | FALSE |  |
| 22:2 CE | Down | 0.00198 | FALSE |  |
| 22:4 CE | Down | 0.30531 | FALSE |  |
| 22:5 CE | Down | 0.09488 | FALSE |  |
| 22:6 CE | Down | 0.44858 | FALSE |  |
| DG 32:0; DG(16:0/16:0) | Down | 0.97473 | FALSE |  |
| DG 32:1; DG(16:0/16:1) | Down | 0.00107 | FALSE |  |
| DG 34:0; DG(16:0/18:0) | Down | 1 | FALSE |  |
| DG 34:1; DG(16:0/18:1) | Up | 0.81818 | FALSE |  |
| DG 36:0; DG(16:0/20:0) | Down | 0.76193 | FALSE |  |
| DG 36:1; DG(18:0/18:1) | Down | 0.05697 | FALSE |  |
| DG 36:2; DG(18:1/18:1) | Down | 0.31736 | FALSE |  |
| PC 28:0; PC(14:0/14:0) | Up | 0.51622 | FALSE |  |
| PC 30:0; PC(14:0/16:0) | Up | 0.01482 | FALSE |  |
| PC 30:1; PC(14:0/16:1) | Down | 0.00789 | FALSE |  |
| PC 31:0; PC(15:0/16:0) | Up | **0.00001** | **TRUE** |  |
| PC 32:0; PC(16:0/16:0) | Up | 0.01499 | FALSE |  |
| PC 32:0; PC(16:0/16:0).1 | Up | 0.0525 | FALSE |  |
| PC 32:1; PC(16:0/16:1) | Up | 0.2625 | FALSE | √[[1](#_ENREF_1)] |
| PC 32:2; PC(16:0/16:2) | Down | 0.03085 | FALSE | √ [[1](#_ENREF_1)] |
| PC 32:2; PC(16:1/16:1) | Down | **0.00003** | **TRUE** |  |
| PC 32:3; PC(16:1/16:2) | Up | 0.60695 | FALSE | √ [[2](#_ENREF_2)] |
| PC 32:4; PC(16:1/16:3) | Down | 0.22501 | FALSE |  |
| PC 33:1; PC(16:0/17:1) | Up | 0.1773 | FALSE |  |
| PC 33:1; PC(16:0/17:1).1 | Up | 0.41189 | FALSE |  |
| PC 33:1; PC(16:0/17:1).2 | Up | 0.24026 | FALSE |  |
| PC 33:1; PC(16:0/17:1).3 | Up | 0.51488 | FALSE |  |
| PC 33:2; PC(16:1/17:1) | Up | 0.39051 | FALSE |  |
| PC 34:1; PC(16:0/18:1) | Down | 0.0254 | FALSE |  |
| PC 34:2; PC(16:1/18:1) | Down | 0.34691 | FALSE | √ [[1](#_ENREF_1)] |
| PC 34:2; PC(16:1/18:1).1 | Down | **0.00002** | **TRUE** |  |
| PC 34:2; PC(17:1/17:1) | Down | 0.00216 | FALSE |  |
| PC 34:3; PC(16:1/18:2) | Down | 0.00216 | FALSE | √ [[2](#_ENREF_2)] |
| PC 34:4; PC(16:1/18:3) | Down | 0.06846 | FALSE | √ [[2](#_ENREF_2)] |
| PC 34:4; PC(18:1/16:3) | Up | 0.09307 | FALSE |  |
| PC 35:1; PC(17:0/18:1) | Up | 0.22674 | FALSE |  |
| PC 35:2; PC(17:1/18:1) | Down | 0.09307 | FALSE |  |
| PC 36:1; PC(18:0/18:1) | Down | 0.18791 | FALSE |  |
| PC 36:2; PC(18:1/18:1) | Down | 0.00499 | FALSE |  |
| PC 36:2; PC(18:1/18:1).1 | Down | 0.004 | FALSE |  |
| PC 36:3; PC(15:1/21:2) | Up | 0.30952 | FALSE | √[[1](#_ENREF_1), [2](#_ENREF_2)] |
| PC 36:3; PC(18:1/18:2) | Up | 0.39394 | FALSE |  |
| PC 36:4; PC(16:0/20:4) | Down | 0.17654 | FALSE | √[[1](#_ENREF_1), [2](#_ENREF_2)] |
| PC 36:4; PC(16:0/20:4).1 | Down | 0.6284 | FALSE |  |
| PC 36:4; PC(18:1/18:3) | Down | 0.01928 | FALSE |  |
| PC 36:5; PC(16:1/20:4) | Down | **0.00036** | **TRUE** | √ [[2](#_ENREF_2)] |
| PC 37:3; PC(18:1/19:2) | Down | 0.09307 | FALSE |  |
| PC 38:2; PC(18:1/20:1) | Down | 0.00955 | FALSE |  |
| PC 38:4; PC(18:0/20:4) | Down | **0.00003** | **TRUE** | √[[1](#_ENREF_1), [2](#_ENREF_2)] |
| PC 38:4; PC(18:0/20:4).1 | Down | 0.27543 | FALSE |  |
| PC 38:4; PC(18:0/20:4).2 | Up | 0.39394 | FALSE |  |
| PC 38:4; PC(18:1/20:3) | Down | **0** | **TRUE** |  |
| PC 38:5; PC(18:1/20:4) | Down | **0.00009** | **TRUE** | √ [[1](#_ENREF_1), [2](#_ENREF_2)] |
| PC 38:6; PC(16:0/22:6) | Down | 0.1031 | FALSE | √ [[1](#_ENREF_1), [2](#_ENREF_2)] |
| PC 38:6; PC(16:0/22:6).1 | Up | 0.60242 | FALSE |  |
| PE 32:1; PE(16:0/16:1) | Down | 0.01184 | FALSE |  |
| PE 32:1; PE(16:0/16:1).1 | Down | 0.77345 | FALSE |  |
| PE 32:2; PE(16:1/16:1) | Down | 0.02293 | FALSE |  |
| PE 34:1; PE(18:0/16:1) | Down | **0.00073** | **TRUE** |  |
| PE 34:3; PE(16:1/18:2) | Down | 0.02002 | FALSE |  |
| PE 35:1; PE(17:0/18:1) | Down | 0.50164 | FALSE |  |
| PE 36:1; PE(18:0/18:1) | Down | 0.03876 | FALSE |  |
| PE 36:2; PE(18:0/18:2) | Up | 1 | FALSE |  |
| PE 36:3; PE(18:1/18:2) | Down | 0.58874 | FALSE |  |
| PE 36:3; PE(19:1/17:2) | Down | 0.03142 | FALSE |  |
| PE 36:4; PE(16:0/20:4) | Down | 0.97196 | FALSE |  |
| PE 36:4; PE(18:1/18:3) | Down | 0.06494 | FALSE |  |
| PE 36:5; PE(14:0/22:5) | Down | 0.81818 | FALSE |  |
| PE 36:5; PE(16:0/20:5) | Down | 0.02229 | FALSE |  |
| PE 36:5; PE(16:0/20:5).1 | Down | 0.02264 | FALSE |  |
| PE 37:3; PE(19:0/18:3) | Up | 0.24026 | FALSE |  |
| PE 38:1; PE(20:0/18:1) | Down | 0.31925 | FALSE |  |
| PE 38:3; PE(18:0/20:3) | Up | 0.24026 | FALSE |  |
| PE 38:4; PE(18:0/20:4) | Up | 0.17965 | FALSE |  |
| PE 38:4; PE(18:1/20:3) | Down | 0.60962 | FALSE |  |
| PE 38:4; PE(18:1/20:3).1 | Up | 0.81818 | FALSE |  |
| PE 38:4; PE(18:1/20:3).2 | Down | 0.42392 | FALSE |  |
| PE 38:5; PE(14:1/24:4) | Down | 0.17297 | FALSE |  |
| PE 38:5; PE(18:0/20:5) | Up | 0.58874 | FALSE |  |
| PE 38:5; PE(18:1/20:4) | Down | 0.05665 | FALSE |  |
| PE 38:6; PE(18:1/20:5) | Down | 0.48485 | FALSE |  |
| PE 40:1; PE(22:0/18:1) | Up | 0.19301 | FALSE |  |
| PE 40:2; PE(18:1/22:1) | Up | 0.17965 | FALSE |  |
| PE 40:2; PE(19:1/21:1) | Down | 0.57609 | FALSE |  |
| PE 40:4; PE(18:0/22:4) | Down | 0.24026 | FALSE |  |
| PE 40:6; PE(18:1/22:5) | Down | 1 | FALSE |  |
| PE 40:7; PE(18:1/22:6) | Up | 0.48485 | FALSE |  |
| PE 42:1; PE(24:0/18:1) | Up | 0.52674 | FALSE |  |
| PE 42:4; PE(18:2/24:2) | Down | 0.97596 | FALSE |  |
| PE 44:5; PE(18:1/26:4) | Down | 0.71835 | FALSE |  |
| PG 34:1; PG(16:0/18:1) | Up | 0.30952 | FALSE |  |
| PPC 18:0; PC(P-14:0/4:0) | Up | 0.24026 | FALSE |  |
| PPC 32:0; PC(P-14:0/18:0) | Down | 0.04955 | FALSE |  |
| PPC 32:2; PC(P-14:0/18:2) | Down | 0.19199 | FALSE |  |
| PPC 33:5; PC(P-15:0/18:5) | Up | 0.48485 | FALSE |  |
| PPC 34:0; PC(P-14:0/20:0) | Down | 0.66845 | FALSE |  |
| PPC 36:0; PC(P-14:0/22:0) | Down | 0.34264 | FALSE |  |
| PPC 36:0; PC(P-14:0/22:0).1 | Down | 0.1083 | FALSE |  |
| PPE 32:1; PE(P-16:0/16:1) | Down | 0.07885 | FALSE |  |
| PPE 34:1; PE(P-16:0/18:1) | Down | 0.09345 | FALSE | √[[1](#_ENREF_1)] |
| PPE 34:2; PE(P-16:0/18:2) | Down | 0.59617 | FALSE |  |
| PPE 34:2; PE(P-16:0/18:2).1 | Down | 0.1138 | FALSE |  |
| PPE 35:0; PE(P-19:0/16:0) | Up | 0.00439 | FALSE |  |
| PPE 36:1; PE(P-18:0/18:1) | Up | 0.0059 | FALSE |  |
| PPE 36:2; PE(P-16:0/20:2) | Up | 0.54481 | FALSE |  |
| PPE 36:3; PE(P-16:0/20:3) | Up | 0.96104 | FALSE |  |
| PPE 36:3; PE(P-16:0/20:3).1 | Down | 0.59053 | FALSE |  |
| PPE 36:3; PE(P-16:0/20:3).2 | Down | 0.00614 | FALSE |  |
| PPE 36:4; PE(P-16:0/20:4) | Up | 0.91722 | FALSE |  |
| PPE 36:4; PE(P-16:0/20:4).1 | Up | 0.01108 | FALSE |  |
| PPE 37:1; PE(P-19:0/18:1) | Down | 0.03915 | FALSE |  |
| PPE 37:2; PE(P-16:0/21:2) | Down | 0.00624 | FALSE |  |
| PPE 37:4; PE(P-17:0/20:4) | Up | 0.07707 | FALSE |  |
| PPE 37:4; PE(P-17:0/20:4).1 | Up | 0.0393 | FALSE |  |
| PPE 38:3; PE(P-16:0/22:3) | Up | 0.69913 | FALSE |  |
| PPE 38:3; PE(P-16:0/22:3).1 | Down | 0.09307 | FALSE |  |
| PPE 38:4; PE(P-16:0/22:4) | Down | 0.00793 | FALSE |  |
| PPE 38:4; PE(P-16:0/22:4).1 | Down | 0.13839 | FALSE |  |
| PPE 38:4; PE(P-18:0/20:4) | Up | 0.05059 | FALSE |  |
| PPE 38:5; PE(P-16:0/22:5) | Up | 0.22924 | FALSE |  |
| PPE 38:5; PE(P-16:0/22:5).1 | Down | 0.42312 | FALSE |  |
| PPE 38:5; PE(P-18:0/20:5) | Up | 0.00112 | FALSE |  |
| PPE 38:6; PE(P-16:0/22:6) | Down | 0.42125 | FALSE | √[[1](#_ENREF_1)] |
| PPE 39:2; PE(P-18:0/21:2) | Down | 0.01745 | FALSE |  |
| PPE 39:5; PE(P-17:0/22:5) | Up | 0.01045 | FALSE |  |
| PPE 40:5; PE(P-18:0/22:5) | Up | 0.03284 | FALSE |  |
| PPE 40:6; PE(P-18:0/22:6) | Up | 0.36022 | FALSE |  |
| SM 34:1; SM(d14:0/20:1) | Up | 0.27796 | FALSE |  |
| SM 36:4; SM(d15:3/21:1) | Down | 0.33245 | FALSE |  |
| TG 42:0; TG(12:0/14:0/16:0) | Up | 0.70104 | FALSE |  |
| TG 44:0; TG(14:0/14:0/16:0) | Up | **0.00074** | **TRUE** |  |
| TG 44:1; TG(12:0/14:0/18:1) | Up | 0.01132 | FALSE |  |
| TG 45:0; TG(14:0/15:0/16:0) | Up | 0.55026 | FALSE |  |
| TG 46:0; TG(14:0/16:0/16:0) | Up | 0.00216 | FALSE |  |
| TG 46:1; TG(14:0/16:0/16:1) | Up | 0.00363 | FALSE |  |
| TG 46:2; TG(14:1/16:0/16:1) | Up | 0.65501 | FALSE |  |
| TG 47:0; TG(15:0/16:0/16:0) | Up | 0.02597 | FALSE |  |
| TG 47:1; TG(15:0/16:0/16:1) | Up | 0.01067 | FALSE |  |
| TG 48:0; TG(14:0/16:0/18:0) | Up | **0.00068** | **TRUE** |  |
| TG 48:1; TG(14:0/16:0/18:1) | Up | 0.00216 | FALSE |  |
| TG 48:2; TG(14:0/16:1/18:1) | Up | 0.10934 | FALSE |  |
| TG 48:3; TG(14:1/16:1/18:1) | Down | 0.09419 | FALSE |  |
| TG 49:0; TG(16:0/16:0/17:0) | Up | 0.04658 | FALSE |  |
| TG 49:1; TG(15:0/16:0/18:1) | Up | **0.00033** | **TRUE** |  |
| TG 49:2; TG(16:0/16:1/17:1) | Up | 0.005 | FALSE |  |
| TG 50:0; TG(16:0/16:0/18:0) | Up | 0.22993 | FALSE |  |
| TG 50:1; TG(16:0/16:0/18:1) | Up | 0.00131 | FALSE |  |
| TG 50:2; TG(16:0/16:1/18:1) | Up | 0.00794 | FALSE |  |
| TG 50:3; TG(16:1/16:1/18:1) | Up | 0.69913 | FALSE |  |
| TG 51:1; TG(16:0/17:0/18:1) | Up | **0.00025** | **TRUE** |  |
| TG 51:2; TG(16:0/17:1/18:1) | Up | **0.00009** | **TRUE** |  |
| TG 51:3; TG(16:1/17:1/18:1) | Up | 0.08585 | FALSE |  |
| TG 52:0; TG(16:0/18:0/18:0) | Up | 0.51121 | FALSE |  |
| TG 52:1; TG(16:0/18:0/18:1) | Up | 0.01089 | FALSE |  |
| TG 52:2; TG(16:0/18:1/18:1) | Up | 0.023 | FALSE |  |
| TG 52:3; TG(16:0/18:1/18:2) | Up | 0.27088 | FALSE |  |
| TG 52:4; TG(16:1/18:1/18:2) | Down | 0.24026 | FALSE |  |
| TG 53:1; TG(17:0/18:0/18:1) | Up | **0.00021** | **TRUE** |  |
| TG 53:2; TG(17:0/18:1/18:1) | Up | 0.00216 | FALSE |  |
| TG 53:3; TG(17:1/18:1/18:1) | Up | 0.00216 | FALSE |  |
| TG 53:4; TG(17:0/17:2/19:2) | Up | 0.6368 | FALSE |  |
| TG 53:5; TG(17:1/17:2/19:2) | Down | 0.91079 | FALSE |  |
| TG 54:1; TG(18:0/18:0/18:1) | Up | 0.04103 | FALSE |  |
| TG 54:2; TG(16:0/18:1/20:1) | Up | 0.05632 | FALSE |  |
| TG 54:3; TG(18:0/18:1/18:2) | Up | 0.22055 | FALSE |  |
| TG 54:4; TG(18:1/18:1/18:2) | Down | 0.36061 | FALSE |  |
| TG 54:5; TG(18:1/18:2/18:2) | Down | 0.14207 | FALSE |  |
| TG 54:6; TG(16:0/18:2/20:4) | Up | **0.00044** | **TRUE** |  |
| TG 54:6; TG(16:1/18:1/20:4) | Up | 0.1975 | FALSE |  |
| TG 54:7; TG(18:1/18:3/18:3) | Up | 0.00167 | FALSE |  |
| TG 55:2; TG(18:0/18:1/19:1) | Up | 0.01465 | FALSE |  |
| TG 55:3; TG(18:1/18:1/19:1) | Up | 0.07662 | FALSE |  |
| TG 55:4; TG(17:0/17:0/21:4) | Up | 0.68449 | FALSE |  |
| TG 55:5; TG(17:0/17:0/21:5) | Down | 0.50138 | FALSE |  |
| TG 55:6; TG(17:0/17:2/21:4) | Up | 0.00634 | FALSE |  |
| TG 55:7; TG(17:1/17:1/21:5) | Up | **0.0003** | **TRUE** |  |
| TG 56:2; TG(18:0/18:1/20:1) | Up | 0.19348 | FALSE |  |
| TG 56:3; TG(18:1/18:1/20:1) | Up | 0.7363 | FALSE |  |
| TG 56:4; TG(18:1/18:2/20:1) | Down | 0.81818 | FALSE |  |
| TG 56:5; TG(16:0/18:1/22:4) | Down | 0.09692 | FALSE |  |
| TG 56:6; TG(16:0/18:1/22:5) | Up | 0.01171 | FALSE |  |
| TG 56:7; TG(16:0/18:1/22:6) | Up | 0.00121 | FALSE |  |
| TG 56:8; TG(16:1/18:1/22:6) | Up | 0.01345 | FALSE |  |
| TG 57:3; TG(18:1/19:1/20:1) | Up | 0.00149 | FALSE |  |
| TG 57:4; TG(18:1/18:1/21:2) | Up | 0.19748 | FALSE |  |
| TG 57:5; TG(17:0/19:1/21:4) | Down | 0.36292 | FALSE |  |
| TG 57:8; TG(19:2/19:3/19:3) | Up | **0.00037** | **TRUE** |  |
| TG 58:3; TG(18:1/18:1/22:1) | Up | 0.78996 | FALSE |  |
| TG 58:4; TG(18:1/20:1/20:2) | Down | 0.28471 | FALSE |  |
| TG 58:5; TG(18:0/18:1/22:4) | Down | 0.00418 | FALSE |  |
| TG 58:6; TG(18:0/18:1/22:5) | Up | 0.02597 | FALSE |  |
| TG 58:7; TG(18:1/18:1/22:5) | Up | 0.02597 | FALSE |  |
| TG 58:7; TG(18:1/18:1/22:5).1 | Up | 0.02597 | FALSE |  |
| TG 58:8; TG(18:1/18:1/22:6) | Up | 0.00989 | FALSE |  |
| TG 60:11; TG(16:0/22:5/22:6) | Up | 0.07811 | FALSE |  |
| TG 60:5; TG(18:1/20:1/22:3) | Down | 0.06494 | FALSE |  |
| TG 60:6; TG(18:1/20:1/22:4) | Down | 0.41707 | FALSE |  |
| TG 60:7; TG(18:1/20:3/22:3) | Up | 0.00216 | FALSE |  |
| TG 60:8; TG(18:1/20:1/22:6) | Up | **0.00026** | **TRUE** |  |

1. Yu, Y., et al., *A lipidomics investigation of the induced hypoxia stress on HeLa cells by using MS and NMR techniques.* Mol Biosyst, 2014. **10**(4): p. 878-90.

2. Schug, Z.T., et al., *Acetyl-CoA synthetase 2 promotes acetate utilization and maintains cancer cell growth under metabolic stress.* Cancer cell, 2015. **27**(1): p. 57-71.
