# Supplementary figures and images for: Cancer cell lipid class homeostasis is altered under nutrient-deprivation but stable under hypoxia
Source: BMC Cancer. 2019 May 28;19:501. doi: 10.1186/s12885-019-5733-y (PMC6537432; doi:10.1186/s12885-019-5733-y)

## Slide 1
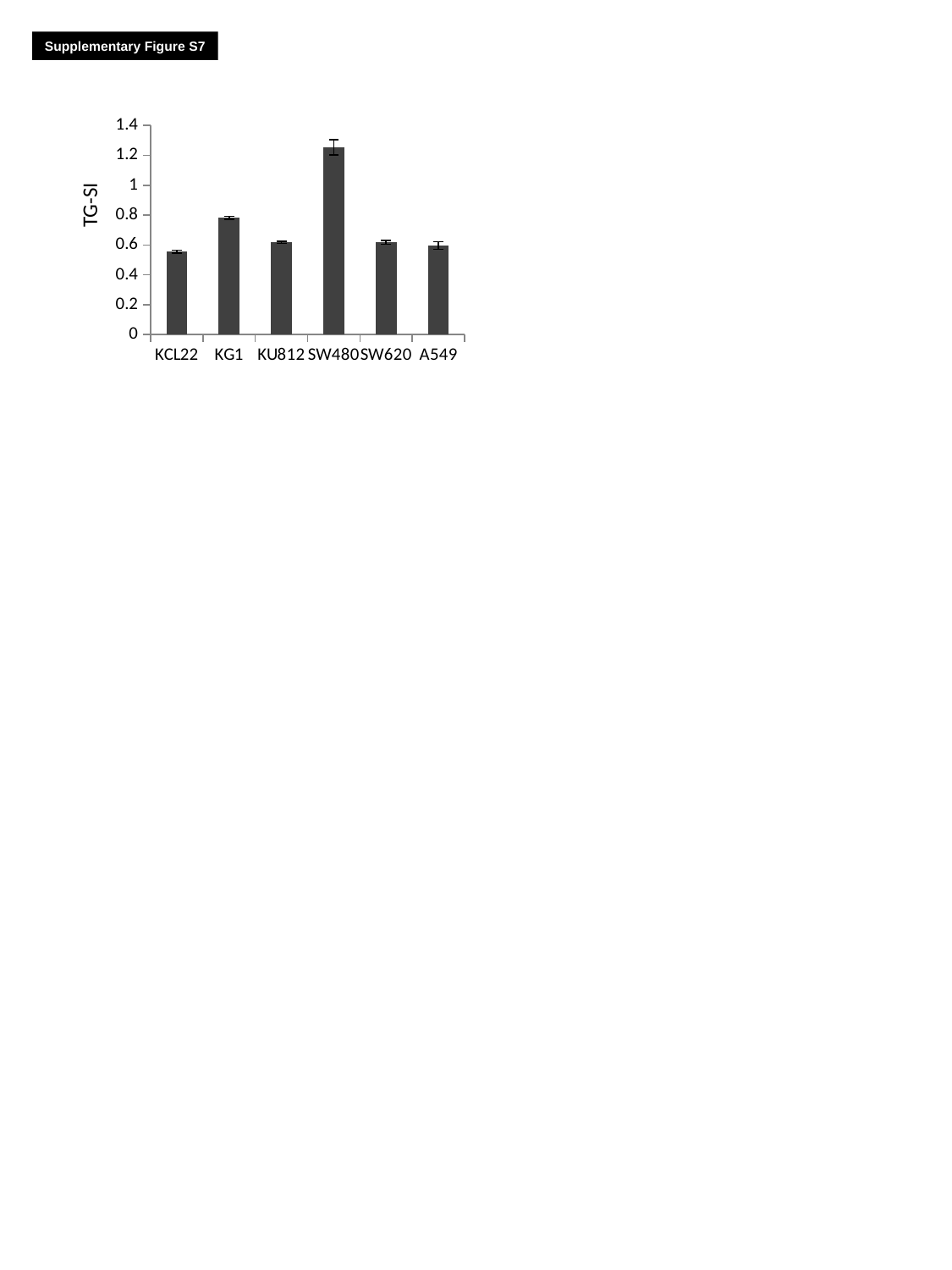

Supplementary Figure S7
### Chart
| Category | TG SI |
|---|---|
| KCL22 | 0.5542118525534032 |
| KG1 | 0.7813586652959523 |
| KU812 | 0.6173115001962924 |
| SW480 | 1.252760605080416 |
| SW620 | 0.6173115001962924 |
| A549 | 0.5963182402393864 |TG-SI

Supplement: Supplementary file 9 — Figure S7. Baseline saturation indices of TGs in KCL22 (Leukemia), KG1 (Leukemia), KU812 (Leukemia), SW480 (Colon cancer), SW620 (Colon cancer), A549 (Lung Cancer). (PPTX 99 kb) [file 12885_2019_5733_MOESM9_ESM.pptx]

## Slide 1
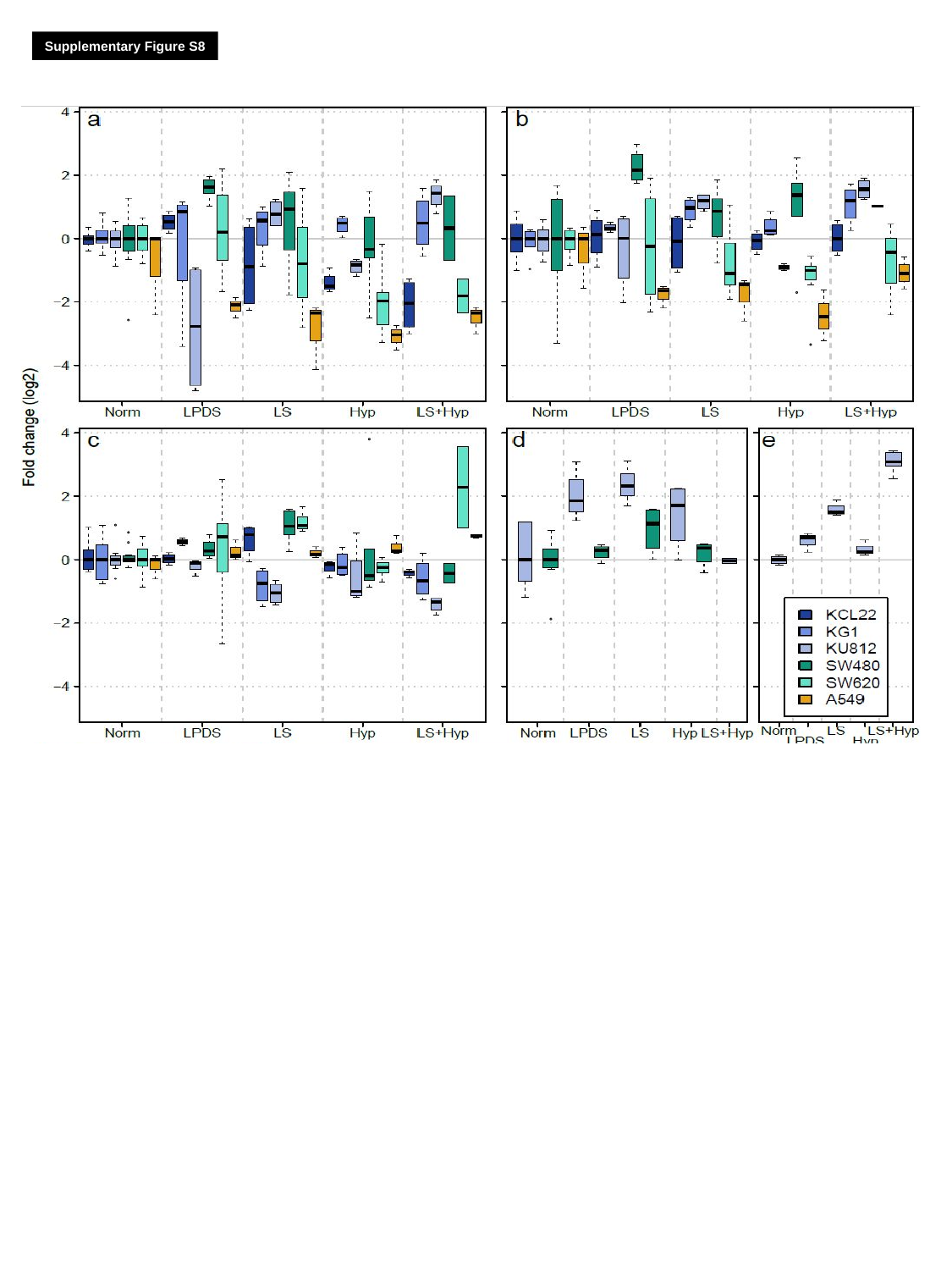

Supplementary Figure S8

Supplement: Supplementary file 10 — Figure S8. Effect of metabolic stress on expression of selected genes from de novo lipid synthesis or lipid uptake/degradation pathways in different cancer cell lines. Box plots showing log2 transformed and median normalized values for (a) FASN (b) HMGCR (c) MGLL expression levels in KCL22 (Leukemia), KG1 (Leukemia), KU812 (Leukemia), SW480 (Colon cancer), SW620 (Colon cancer) and A549 (Lung Cancer). (d) LPL expression level in KU812 (Leukemia) and SW480 (Colon cancer) cells. (e) CD36 expression level in KU812 (Leukemia) cells. Cells were cultivated (48 hours) under lipoprotein deficient medium (LPDS serum), low-serum (LS) medium (2% serum), hypoxia (2% O2), or hypoxia in combination with LS medium. The levels of the different transcripts were measured in 3 to 6 samples by qPCR. The results show the distribution of corresponding transcripts relative to GAPDH, with the box indicating the 25th–75th percentiles, with the median indicated. line. The whiskers show the range. Data were normalized to the median expression level of the given transcript under normal conditions for the respective cell line. (PPTX 218 kb) [file 12885_2019_5733_MOESM10_ESM.pptx]
